# Highlighting of the interactions of MYD88 and NFKB1 SNPs in rats resistant to decompression sickness: toward an autoimmune response

**DOI:** 10.3389/fphys.2023.1253856

**Published:** 2023-08-17

**Authors:** Nicolas Vallée, Emmanuel Dugrenot, Anne-Virginie Desruelle, Simone Richard, Stéphane Coupé, Céline Ramdani, Régis Guieu, Jean-Jacques Risso, Sandrine Gaillard, François Guerrero

**Affiliations:** ^1^ Institut de Recherche Biomédicale des Armées, Equipe de Recherche Subaquatique Opérationnelle, Toulon, France; ^2^ Université de Brest Occidentale, ORPHY, IBSAM, Brest, France; ^3^ Université de Toulon, La Garde, France; ^4^ Université d’Aix-Marseille, Marseille, France

**Keywords:** 3′UTR, 5′UTR, autoimmune, decompression sickness, dive, hyperoxia (oxygen)

## Abstract

Decompression sickness (DCS) with neurological disorders includes an inappropriate inflammatory response which degenerates slowly, even after the disappearance of the bubbles. There is high inter-individual variability in terms of the occurrence of DCS that could have been mastered by the selection and then the breeding of DCS-resistant rats. We hypothesized the selection of single-nucleotide polymorphisms (SNPs) linked to autoimmunity operated upon a generation of a DCS-resistant strain of rats. We used the candidate gene approach and targeted SNPs linked to the signaling cascade that directly regulates inflammation of innate immunity transiting by the Toll-like receptors. Twenty candidate SNPs were investigated in 36 standard rats and 33 DCS-resistant rats. For the first time, we identify a diplotype (i.e., with matched haplotypes)—when coinherited—that strengthens protection against DCS, which is not strictly homozygous and suggests that a certain tolerance may be considered. We deduced an ideal haplotype of six variants from it (MyD88_50-T, _49-A, _97-C coupled to NFKB_85-T, _69-T, _45-T) linked to the resistant phenotype. Four among the six identified variants are located in pre- and/or post-transcriptional areas regulating MyD88 or NFKB1 expression. Because of missense mutations, the other two variants induce a structural change in the NFKB1 protein complex including one damage alteration according to the Missense3D algorithm. In addition to the MyD88/NFKB1 haplotype providing rats with a strong resistance to DCS, this also highlights the importance that the immune response, here linked to the genetic heritage, can have in the development of DCS and offer a new perspective for therapeutic strategies.

## Introduction

When diving, the gases breathed in through the regulator are dissolved in the body tissues progressively during the descent to the seabed. During the decompression phase, they may give rise to the production of bubbles. When bubbles form in excessive quantities in the blood and tissues, symptoms of decompression sickness (DCS) may appear (Bert, 1978). Conventionally, it is acknowledged that the quantity of venous bubbles has a positive correlation to the risk of DCS (Sawatzky and Nishi, 1991; Brubakk and Neumann, 2003). However, there is high intra- and inter-individual variability in terms of bubble formation and occurrence of DCS for the same dive profile.

Among the multiplicity of identified factors that can lead to DCS, such as age (Ardestani et al., 2015), weight (Pollock, 2007), or the presence of a permeable foramen ovale (Honěk et al., 2019), it also seems that inherited characteristics, such as sex (Hagberg and Ornhagen, 2003; Buzzacott et al., 2014), can be much influential (Lautridou et al., 2017). It has also been suggested that the digestive tract, through the contribution of the intestinal microbiota, could have an influence on the occurrence of DCS (Kayar et al., 1998; Kayar et al., 2001; Kayar and Fahlman, 2001; de Maistre et al., 2016a; de Maistre et al., 2016b; de Maistre et al., 2018; de Maistre et al., 2020; Vallee et al., 2021; Desruelle et al., 2022). Interestingly, the composition of the gut microbiota is strongly impacted by the genetic background of the animal strain (Korach-Rechtman et al., 2019). In fact, the heritable component of DCS can be highlighted through the selection and then the breeding of rats resistant to DCS (Lautridou et al., 2017; Lautridou et al., 2020).

DCS includes the inflammatory process that could be countered by fluoxetine (Vallee et al., 2016) or some anti-GPIIb/IIIa (Lambrechts et al., 2018), but not by minocycline (Desruelle et al., 2019), per example. These molecules that interact with immunological responses allowed us to suggest that, in the absence of a well-identified pathogen, we would be facing an autoimmune-like disease, with therefore unexpected responses from the organism.

This study is an extension of previous work (Lautridou et al., 2017; Lautridou et al., 2020), where rats without DCS were selected and bred to create a new generation and then subjected to the same hyperbaric protocol. This procedure was repeated, and the proportion of DCS could be drastically reduced from 65% to 35% in the third generation and 23% in the sixth generation (Lautridou et al., 2017; Lautridou et al., 2020). Moreover, sex-dependent differences were observed in both the gain of resistance to DCS throughout generations and the physiological modifications associated with resistance to DCS. This study investigated the genetic background of the offspring of these selected rats that were not exposed to DCS stress.

The present study is particularly devoted to single-nucleotide polymorphisms (SNPs) linked to innate immunity transiting by the Toll-like receptors (TLRs) and more particularly to the proteins involved in its signaling cascade that directly regulates inflammation. We believe that the selection of such SNPs could be carried out upon generation of DCS-resistant strains of rats.

Here, we studied male and female rats from a strain of rats selected for their resistance to DCS, and we highlight for the first time that certain combinations of MyD88 (innate and adaptive immune signal transduction adapter) and NFKB1—when coinherited—strengthen protection against DCS. The aim is to verify whether this strain of rats resistant to the accident have a genetic signature different from that of the stem generations sensitive to DCS.

## Materials and methods

### Animals and ethical statement

All procedures involving experimental animals follow the 3Rs and complied with European Union rules (Directive 2010/63/EU) and French law (Decree 2013/118). The Ethics Committee of the Université de Bretagne Occidentale approved this study (approval no; APAFIS#10395-2017061909495511). The standard population was composed of 6-week-old Wistar rats (Janvier SAS, Geneste, France). They were housed in an accredited animal care facility, under controlled temperature (22°C ± 1°C) and lighting (12 h of light per day, 6:00 a.m.–6:00 p.m.). They were fed standard rat kibble and water was provided *ad libitum*.

### Animals

Thirty-three DCS-resistant (Res) animals (17 females and 16 males), aged 14 weeks, bred at the university animal house, were used in this study. They were compared to 36 age-matched standard (Std) Wistar rats (18 females and 18 males), i.e., the same as those we used for the founding stock, obtained from the same breeder (Janvier Labs, St Genêts, France). The standard rats were acclimated with the facility for at least 2 weeks. All animals were housed three per cage under controlled temperature (21°C ± 1°C) and lighting (12 h of light per day, 0600–1800) conditions at the university animal housing facility until the day of cecal content harvesting. They were fed standard rat chow and water *ad libitum*.

As a reminder, the DCS-resistant strain of rats is the result of a breeding program described in previous studies (Lautridou et al., 2017; Lautridou et al., 2020). The simulated air dive in a hyperbaric chamber, aimed at selecting resistant rats for breeding, was as follows: compression was carried out at 1 bar min^−1^ up to 10 ATA. The animals remained for 45 min at this depth before starting the decompression at a speed of 1 bar min^−1^, comprising three stops: 5 min at 2.0 ATA, 5 min at 1.6 ATA, and 10 min at 1.3 ATA. After exiting the hyperbaric chamber, the rats were observed for 1 h for symptoms of DCS, including respiratory distress, paralysis, convulsions, and death. Only rats that showed no visible signs of DCS were considered asymptomatic and selected for the breeding program (Lautridou et al., 2017; Lautridou et al., 2020).

This study investigated the genetic background of offspring that were not exposed to DCS stress.

### Biopsy

Animals were anesthetized first by administration of gaseous isoflurane (4.0% in air flow at 2.0 L/min) through a face mask for 5 min before intraperitoneal injection of ketamine (100 mg kg^−1^) and xylazine (10 mg kg^−1^). Anesthesia levels were determined by testing the lack of withdrawal reflexes in response to pinches of the distal hind limbs. The liver biopsy sample was placed in a 1.5-mL Eppendorf tube and kept at −80°C until genetic analysis. Finally, animals were euthanized with a lethal dose of anesthesia.

### DNA extraction and SNP genotyping

DNA for PCR was extracted from cells from liver cells with the NucleoSpin Tissue kit. The recommendations of the suppliers have been followed with a modification during the first washing step. The step was repeated, and the centrifugation time was increased threefold.

The concentration of the extract was adjusted to 10 ng/μL in ultrapure water before amplification.

Real-time PCR was carried out with a LightCycler 480 II (LightCycler 480 Software Release; Roche Diagnostics, Mannheim, Germany) on 2 μL DNA with 8 μL reaction mixture. For the negative controls, water was substituted for the extract. The reaction mixture for SNP amplification contained 1 μL of each forward and reverse SNP primer (Supplementary Data S1) (1 µM final; Eurogentec, Seraing, Belgium), 0.5 µL of each wild type and mutant SNP probe, and 5 μL of an amplification mixture (GoTaq^®^ Probe qPCR Master Mix, Promega, Madison, United States).

Thermocycler settings were as follows: initialization step: 95°C/10 min; (denaturation step: 95°C/10 s; annealing and elongation step: 63°C (or 60°C)/20 s) × 45 cycles. All assays were carried out in duplicate.

### Genetic association analysis

The association study was performed using all SNPs, considered independent loci, or using alleles, considering the gene loci from which SNPs are derived, as independent loci. For the latter, the most likely haplotypes were reconstructed using DNAsp (v6.12.03) (Rozas et al., 2017), using default parameters and 1,000 Markov chain Monte Carlo iterations. Then, we obtained two genotype datasets using either the SNPs or the deduced alleles.

The basic genetic diversity indices were obtained using the online version of Genepop (Rousset, 2008) software (v4.7.5). This software was also used to assess eventual deviations from Hardy–Weinberg equilibrium (F_IS_ and F_ST_) and highlight the most contributing SNPs or alleles to the population/group structure.

Further statistical analyses were performed. Kruskal–Wallis tests supplemented with Dunn’s *post-hoc* (with Bonferroni correction) were carried out in order to verify the existence of differences between the populations by considering the SNPs as being isolated, or as being an integral part of a haplotype or even a diplotype. The association between qualitative variables (Group, SNP, and sex) was studied using multiple correspondence analysis (MCA) conducted from disjunctive table. The MCA was restricted to the categories whose total contribution exceeded 80%. Agglomerative hierarchical clustering (AHC) (dissimilarity; Euclidean distance; Ward’s method) was used to comfort the identification of SNP and haplotypes from normalized data of the principal coordinate of the most contributing axes of the 69 rats. The software was XLSTAT Biomed from Addinsoft. The maximum accepted alpha level was 5%.

## Results

### Genotyping and genetic basis indices

A total of 20 candidate SNPs linked to the immune system were investigated in 36 standard rats and 33 DCS-resistant rats. In these 69 Wistar rats, 16 primers gave rise to amplifications and eight sequences were heterozygous. The only six primers associated to MyD88 and NFKB1 revealed a diversity between resistant and standard rats (Supplementary Data S2) and deserved to be studied in this work. Considering the low number of individuals in these populations but faced with the need to characterize them, we only briefly carried out population genetic statistics.

**FIGURE 1:**
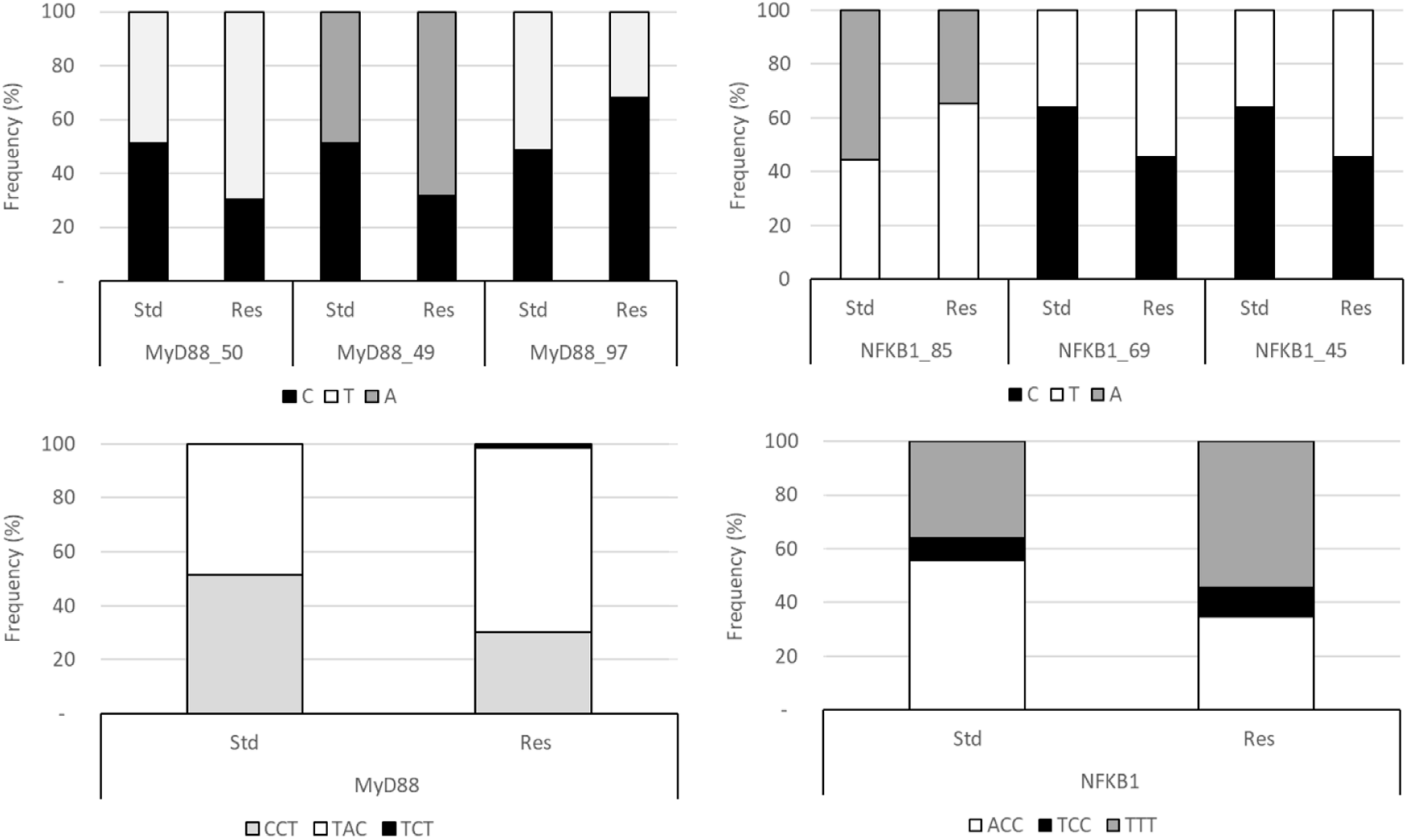
Frequencies of SNP and haplotypes in Res and standard Wistar rats. The MyD88 haplotype is ordered according to the sequence MyD88_50/MyD88_49/MyD88_97 and that of NFKB1 is ordered as the sequence NFK_85/NFK_69/NFK_49.

Therefore, rs1074255550 (MyD88_50), rs106151549 (MyD88_49), rs198397997 (MyD88_97), rs19813385 (NFK_85), rs197284969 (NFK_69), and rs197247545 (NFK_45) Fis estimates were calculated, independently or not, i.e., for the MyD88 haplotype on one hand and the NFKB1 haplotype on the other hand. Allele frequencies are presented in Figure 1 for MyD88 or NFKB1 haplotypes, respectively (not all combinations are shown).

When all SNPs were considered independent, no locus escapes the panmictic model (Table 1), strictly speaking (Chi2 test on Fis), even though NFK_85 has a significant *p*-value. Nonetheless, the NFKB1 haplotype escapes the panmictic model in the standard population, especially in males, probably because of NFK_85 and inbreeding. Interestingly, heterozygosity appears to be restored in the Res population.

**TABLE 1:** Fixation indices and inbreeding coefficient.

	p-value of fis estimates	
Locus	Std	Res
MYD_^50^	**0.7455**	**1**
MyD_49	**0.7455**	**0.6886**
MY^17^5^7^	**0.7455**	**0.6886**
NFK_85	**0.0088**	**0.4559**
NFK_69	**1**	**0.3**
NFK_45	**1**	**0.3**
Chi2	**11.2344**	**7.8796**
Prob	**0.508949**	**0.794467**
All locus (*n* = 6), all populations (*n* = 2)		
Fisher’s method	**Chi2: 19.1139**	
	**Prob: 0.745886**	

	p-value of fis estimates			
Locus	Std_F	Std_M	Res_F	Res_M
MyD_50	**0.6562**	**1**	**0.2604**	**0.2561**
MyD_49	**0.6566**	**1**	**0.2604**	**1**
MyD_97	**0.656E**	**1**	**0.2604**	**1**
NFK_85	**0.6544**	**0.0013**	**0.6244**	**0.5914**
NFK_69	**0.6244**	**0.2542**	**0.3578**	**1**
NFK 45	**0.6244**	**0.2542**	**0.3578**	**1**
Ch12	**5.2555**	**18.7704**	**13.1256**	**3.7751**
Prob	**0.948898**	**0.094223**	**0.359981**	**0.987161**
All loci (*n*), all populations (*n* = 4)				
Fisher’s method			**Chit: 40.9265**	
			**Prob: 0.755476**	

	P-value of fis estimates	
Haplotype	S W	Res
MyD88	**0.7455**	**0.4527**
FKB1	**0.0034**	**0.1275**
Chi2	**11.9491**	**5.7046**
Prob	**0.0177**	**0.222326**
All locus (*n* = 2), all populations (*n* = 2)		
Fisher’s method	**Chi2: 17.6536**	
	**Prob: 0.023979**	

	P-value of fis estimates			
Haplotype	Std_F	Std_M	Res_F	Res_M
yD88	**0.65**	**1**	**0.2604**	**0.1885**
FKB1	**0.28**	**0.0016**	**0.3001**	**0.6657**
Chi2	**3.3417**	**12.8968**	**5.0983**	**4.1507**
Prob	**0.5024**	**0.0118**	**0.2774**	**0.3860**
All locus (*n* = 2), all populations (*n* = 4)				
Fisher’s method		**Chi2: 25.4874**		
		**Prob: 0.061681**		

**FIGURE 2:**
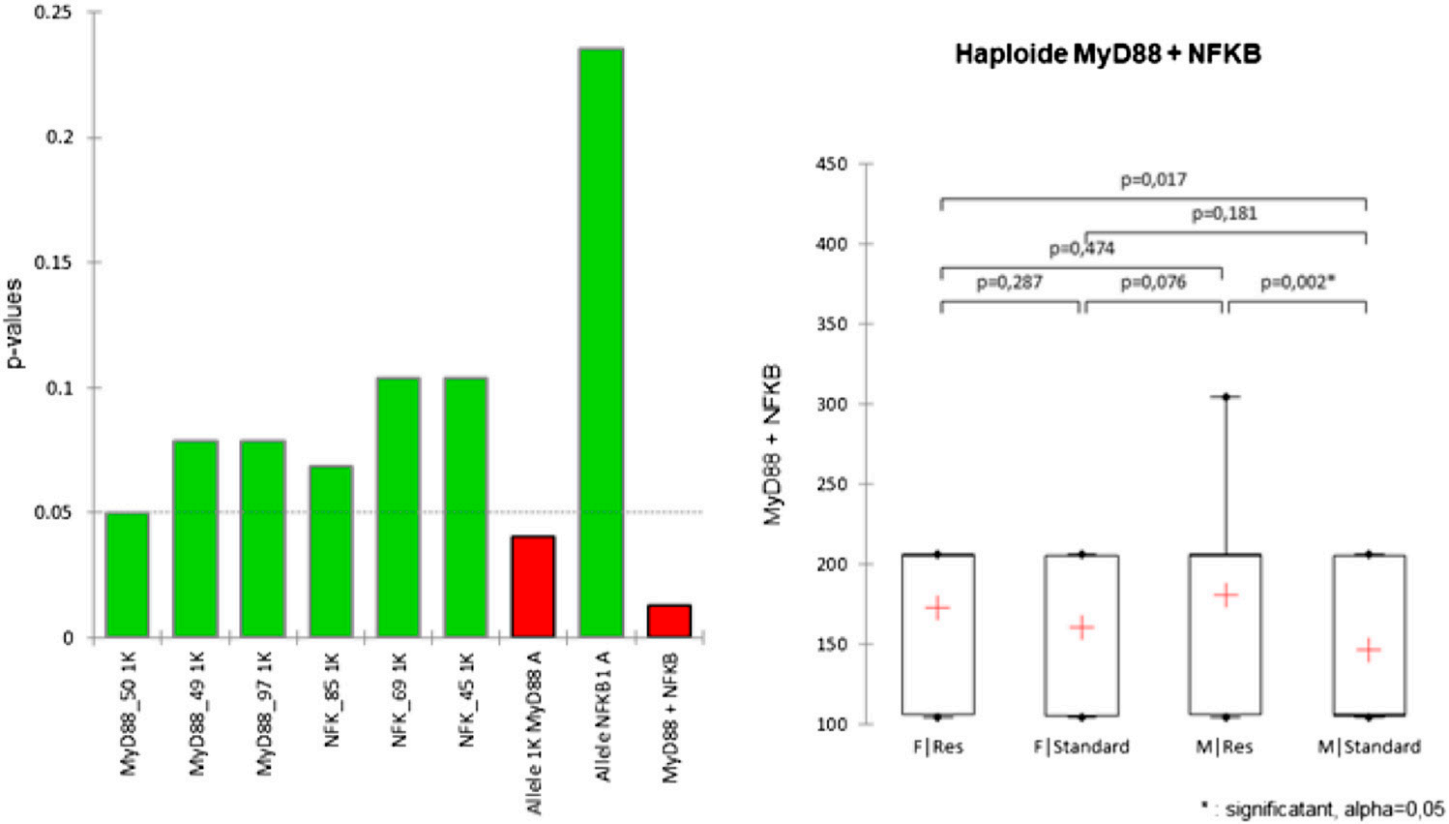
Kruskal–Wallis test conducted on haplotypes of Res and standard Wistar rats (left). *Post*-*hoc* results are shown when significant (right).

**FIGURE 3:**
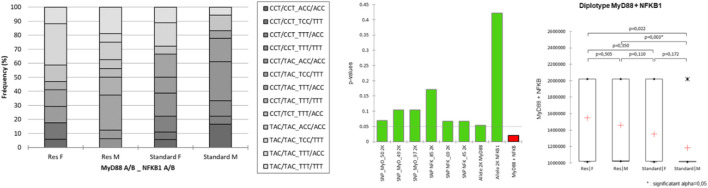
Diplotype analysis. (Left) Frequencies of diplotypes in Res and standard Wistar rats. The MyD88 diplotype is ordered according to the sequence MyD88_50/MyD88_49/MyD88_97 and that of NFKB1 is ordered as the sequence NFKB1_85/NFKB1_69/NFKB1_49 for chromosomes A and B. The diplotype corresponds to the association of haplotypes of a same individual, and the chromosomes are conventionally named A and B, according to the phasing. (Center) Kruskal–Wallis tests conducted on haplotypes of Res and standard Wistar rats. (Right) *Post*-h*oc* result is shown when significant.

Considering these results, Kruskal–Wallis tests supplemented with Dunn’s *post-hoc* (with Bonferroni correction) were carried out in order to verify the existence of differences between the populations by considering the SNPs as being isolated, or as being an integral part of an haplotype (Figure 2) or even a diplotype (Figure 3).

Despite the significance of the Kruskal–Wallis test at the MyD88 haplotype level, *post-hoc* tests show that only the haplotype comprising both the MyD88 and NFKB1 SNPs shows a significant difference in Res M compared to the standard M. No SNPs, taken alone, show any significant difference.

It appears that only the diplotype comprising both the SNPs of MyD88 and NFKB1 shows a significant difference of the Res M compared to the standard M. No pair of SNPs in its diploid form shows any significant difference.

**FIGURE 4:**
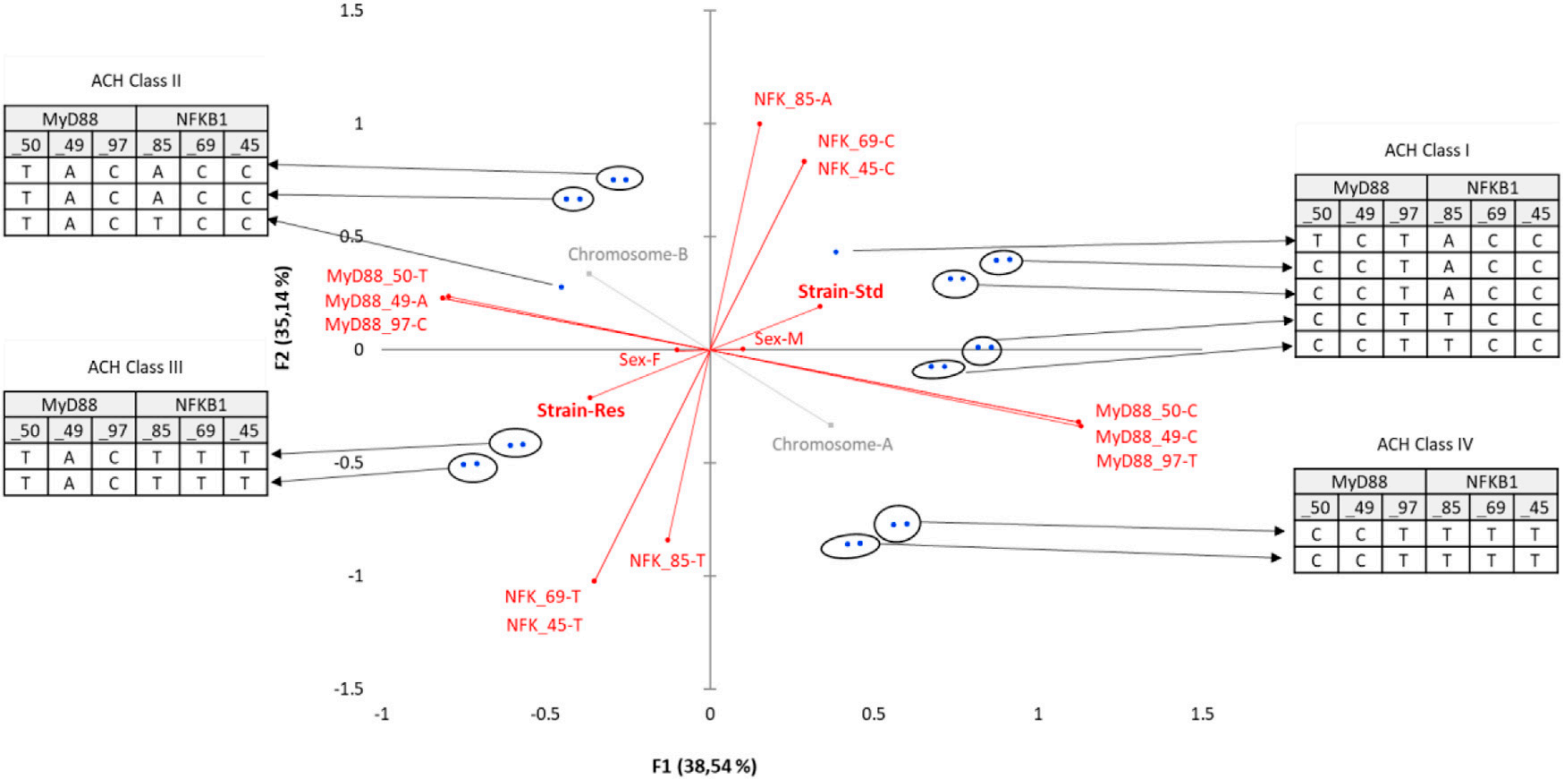
Multiple correspondence analysis (MCA) plot conducted on the SNPs of MyD88 and NFKB1, strain (Standard or Res), sex (male or female), and chromosome (as a supplementary category, A or B). Main categories are displayed in red. Observations are shown in blue. Supplementary variables are shown in gray. The results of the agglomerative hierarchical clustering (ACH), showing the different classes, are reported on the sidelines of the MCA.

MCA was conducted from a full disjunctive table with the criteria as SNPs, population, sex (category), and belonging to one chromosome or another [according to the phasing result, and integrated here as a supplementary category (A or B)] (Figure 4). The axes F1 and F2 explain 73% of the variability. The Res strain is halfway between MyD88_50-T, _49-A, _97-C nucleotides and NFKB_85-T, _69-T, _45-T nucleotides. NFKB-85, slightly distant from Res, does not contribute significantly to the construction of F1. NFKB-85 participates in the construction of F2. F3 accounts for an additional 13% of MCA variability, with an orthogonality of the sex-linked category compared to SNP nucleotides. Actually, sex is the only variable that contributes significantly to the construction of F3, which seems to refute the idea that belonging to a sex would still be a protective factor, in this resistant population and considering these SNPs studied. The position on one chromosome or the other seems to contribute little.

Concretely, the haplotypes of the two clusters present in the lower left quarter of the plot are those that contribute the most to the DCS-resistant phenotype. Identification of the haplotypes of individuals in these two clusters reveals a single sequence of SNPs as follows: MyD88_50-T, _49-A, _97-C coupled to NFKB_85-T, _69-T, _45-T. This result was confirmed by an AHC carried out according to the principal coordinates of the three main axes of the MCA (classes are reported in Figure 4).

Following the evolution of the variances according to the number of classes, a clear inflection point is located in class IV. The division into four classes explains 17% of the intragroup variability. Class III, corresponding to the Res phenotype, contains 38 haplotypes including 14 standard haplotypes, with notably the same sequence of SNPs MyD88_50-T, _49-A, _97-C coupled to NFKB_85-T, _69-T, _45-T. On the contrary, class I, which is close to the standard phenotype, comprises 34 haplotypes including 25 standard haplotypes with a greater variability. Moreover, there is also a pronounced dichotomy for MyD88 between the left and the right side of the MCA, as no variability in the haplotype of MyD88 is observed on the left side, i.e., the Res side (more details in Supplementary Data S3).

**FIGURE 5:**
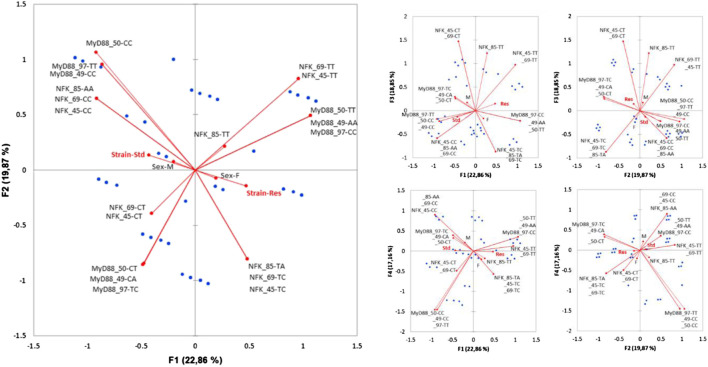
Multiple correspondence analysis (MCA) plots conducted on SNP pairs of MyD88 and NFKB1, strain (standard or Res), and sex (male or female). Observations are shown in blue. Supplementary variables are shown in gray.

A similar MCA (Figure 5) with matched haplotype pairs, i.e., diplotypes, was also carried out.

The axes F1 (22%) and F2 (21%) explain 43% of the variability. Strain only contributes significantly to the F1 construction. The Res strain is halfway between MyD88_50-TT, _49-AA, _97-CC nucleotides and NFKB_85-TA, _69-TC, _45-TC nucleotides. Upon closer examination of the significant variables of the MCA, MyD88 diplotypes remain central, all of its SNPs being significantly contributory to the construction of the F1, F2, and F4 (17%) axes. The NFKB1 SNPs participate significantly in the construction of the F1, F2, and especially F3 (19%) axes. In contrast, sex is only significantly present on F5, which accounts for a lesser contribution (10%) of the MCA.

NFKB_85-TT, which seems to be closer to Res, only significantly contributes to the construction of F3. In the same way, NFKB_69^−ΔΔCT^ and NFKB_45^−ΔΔCT^ participate in the construction of F3 and also F5 (not shown).

**FIGURE 6:**
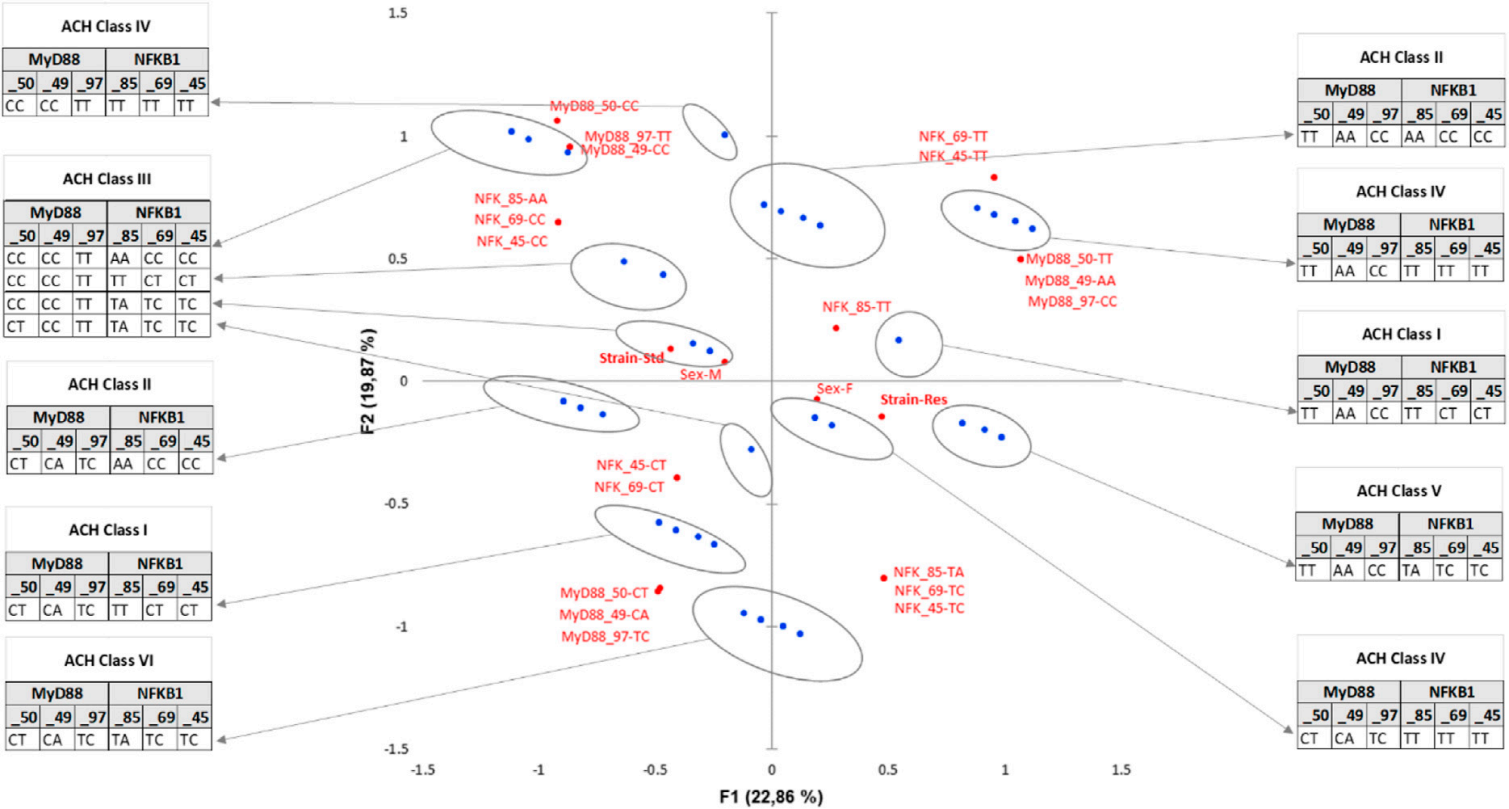
Enriched multiple correspondence analysis plot with agglomerative hierarchical clustering classes. MCA was conducted on the SNP pairs of MyD88 and NFKB1, strain (standard or Res), and sex (male or female). Observations are shown in blue. Supplementary variables are shown in gray. The results of the agglomerative hierarchical clustering (ACH), showing the different classes, are reported on the sidelines of the MCA.

Finally, diplotypes of the cluster comprising nine rats (three standards and six Res) in the lower right quarter of the plot F1 F2 are those that contribute the most to the DCS-resistant phenotype. Identification of the haplotypes of individuals in this cluster reveals such a diplotype MyD88_50-TT, _49-AA, _97-CC coupled to NFKB_85-TA, _69-TC, _45-TC. This result was also seen by the AHC carried out according to the principal coordinates of the five MCA axes (Figure 6). Following the evolution of the variances according to the number of classes, an inflection point is visible in classes V and VI. The division into five classes explains 35% of the intragroup variability, while the division of the population into six classes explains 27% of the variability. The class V corresponds to the Res phenotype in the F1 F2 plot mentioned previously. In this class (V), MyD88 is clearly a homozygote regardless of the SNP, and moreover, it is the same sequence as that identified in the MyD88 haplotype (Figure 4: class IV, MyD88_50-T, _49-A, _97-C). In the diplotype of class V, the expressed NFKB1 SNPs (Figure 6: class IV, NFKB_85-TA, _69-TC, _45-TC) are slightly different from those previously identified in the haplotype. Nevertheless, it is possible to identify the NFKB_85-T, _69-T, _45-T haplotype, the one that was previously close to the Res phenotype (Figure 4: class IV). Furthermore, the NFKB1 diplotype NFKB_85-TT, _69-TT, _45-TT, indicating strict homozygosity, is referred in the ACH class IV, near the Res point of the ACM plot.

## Discussion

Overall, these results show that to have a resistant phenotype, the reference nucleotide of the different SNPs has given way to the substitution nucleotide. The variation of a single SNP does not seem to be decisive for acquiring a resistant phenotype, nor even the rearrangement of a single gene with its SNPs, but rather the combination of two genes studied here, namely, MyD88 and NFKB1, which comprise a total of six SNPs. Without being exclusive, the haplotype ideally linked to the resistant phenotype would be MyD88_50-T, _49-A, _97-C on the chromosome 2 coupled to NFKB_85-T, _69-T, _45-T on chromosome 8 of rats.

The fact that the diplotype selected (i.e., with matched haplotypes) to provide resistance to DCS (MyD88_50-TT, _49-AA, _97-CC, NFKB_85-TA, _69-TC, _45-TC) is not strictly homozygous, suggesting that a certain tolerance may be considered at the level of NFKB1, or that a recessivity or dominance factor may exist. A certain heterozygosity can also find an explanation in the re-introduction during breeding of the DCS-resistant strain of some standard Wistar rats, in order to avoid problems related to consanguinity problems (Lautridou et al., 2020).

**FIGURE 7:**
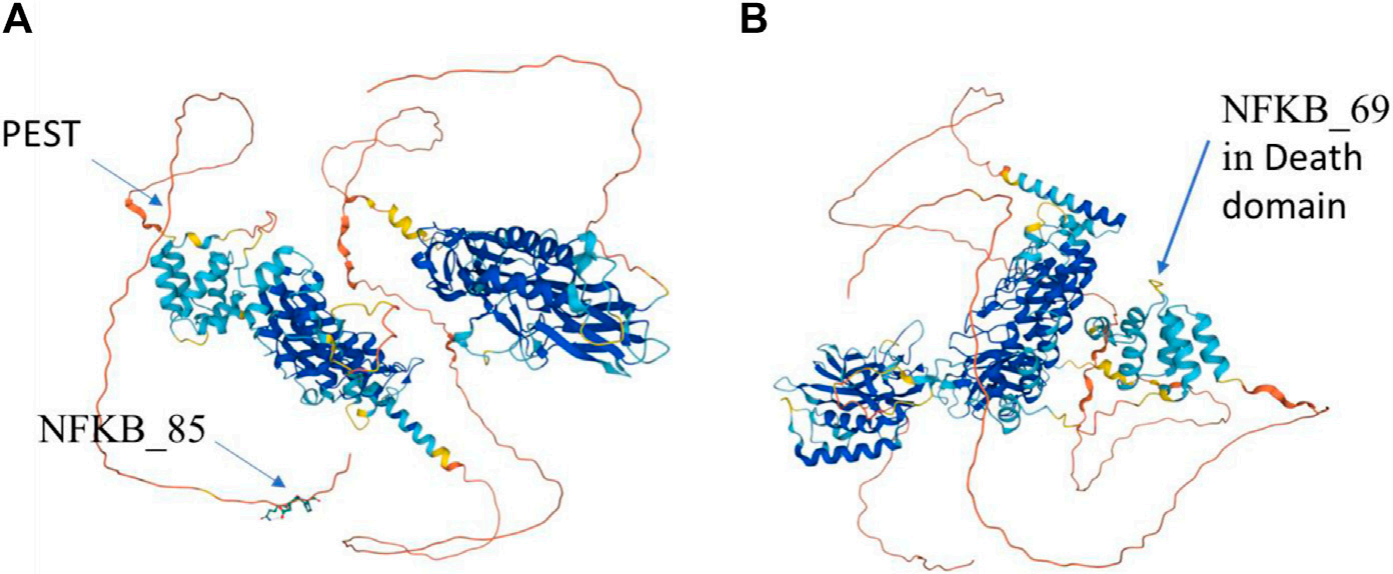
AlphaFold F1LGH2 modeling (Consortium, 2022) of the NFKB1 protein, from two viewpoints, allowing to visualize the impact points of the variants NFKB_85 **(A)** and NFKB_69 **(B)**.

These statements are confirmed, in particular, by the fact that not all Res rats are strictly homozygous. Thus, 4/33 Res rats do not have the MyD88_50-T, _49-A, _97-C haplotype (Figure 7) and five of them do not have the NFKB_85-T, _69-T, _45-T haplotype. Only one of the Res rats showed neither MyD88_50-T, _49-A, _97-C nor NFKB_85-T, _69-T, _45-T. Finally, this also points out one of the limitations of this study, which is only few genetic criteria are assessed, while there are others which are not exclusively of a genetic nature.

### Involvement of SNPs at the transcript level

We researched the Ensembl database (Cunningham et al., 2021) for rats and found that a permutation on MyD88_50 (C>T) or MyD88_49 (C>A) has consequences on the 3′ untranslated transcribed region (3′ UTR) regulating the expression of MyD88, found behind the STOP codon. Concerning the Myd88_97 variant, a nucleotide change (T>C) affects the regulatory region 5′ UTR, which is a segment of the messenger RNA (mRNA) placed upstream of the initiation codon. Please note that this SNP is only described in the context of the ENSRNOT00000096753.1 transcript of the MyD88-204 isoform containing 119 amino acids. The structure of the A0A8I5ZSW8_RAT protein remains poorly described at present.

NFKB1 has two protein forms, namely, NFKB1-201 of 970 amino acids (aa) and another shorter NFKB1-202 of 1,011 aa, both of which will undergo modification in their expression level if the 3′UTR zone undergoes a permutation in NFKB_45 (C>T). Such an SNP would either enhance or repress MyD88 and NFKB1 activations, indirectly. The 5′UTR can regulate the translation of the main coding sequence of the mRNA. The 5′ UTR has been found to interact with proteins relating to metabolism. The 3′-UTR often contains regulatory regions that post-transcriptionally influence gene expression. The 3′-UTR contains both binding sites for regulatory proteins and microRNAs (miRNAs). By binding to specific sites within the 3′-UTR, miRNAs can decrease the gene expression of various mRNAs by either inhibiting translation or directly causing degradation of the transcript. The 3′-UTR also has silencer regions which bind to repressor proteins and will inhibit the expression of the mRNA.

### Involvement of SNPs at the protein level

SNPs NFKB_85 and NFKB_69 cause nonsense mutations at two levels of the NFKB1 protein (Figure 7).

**FIGURE 8:**
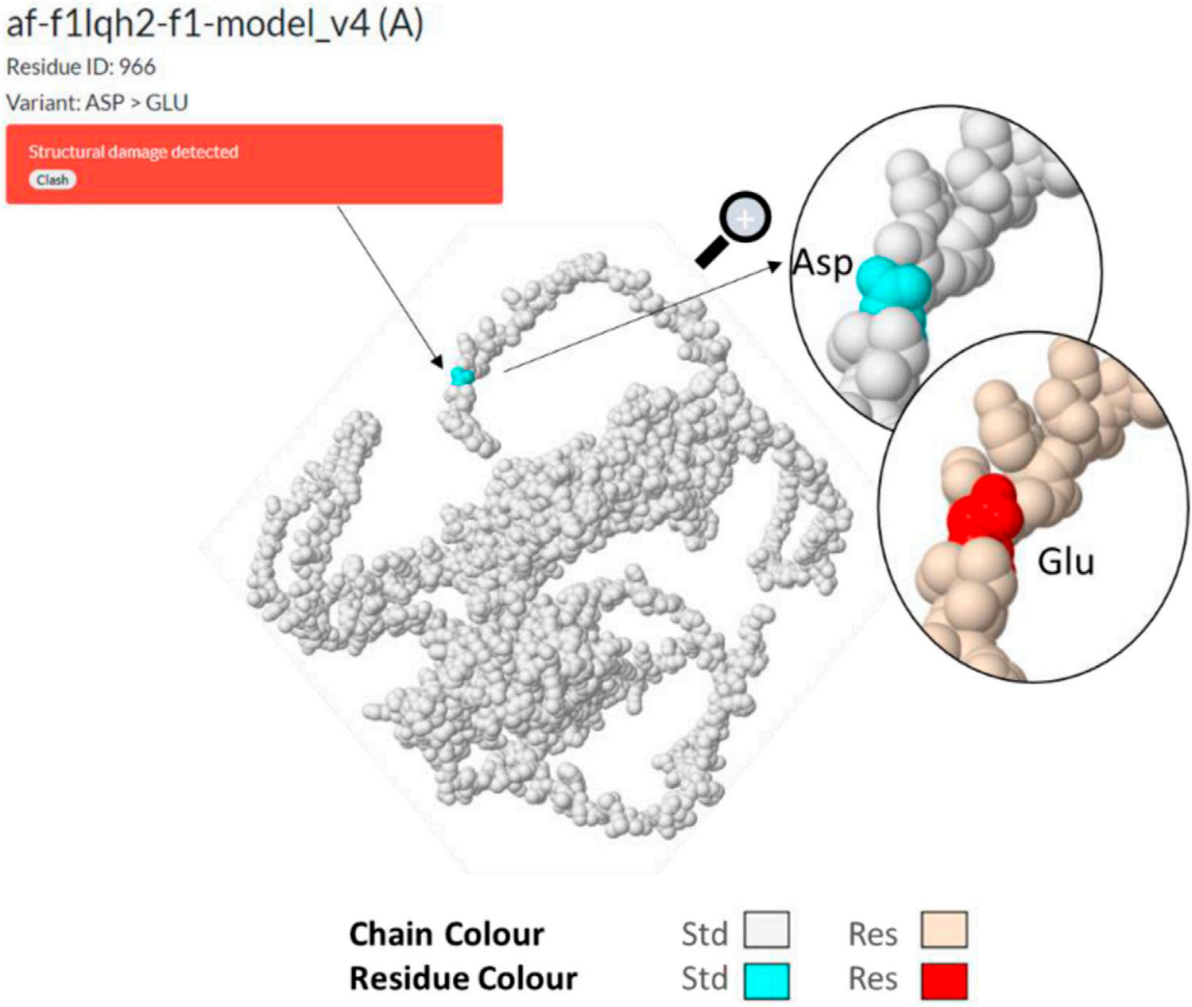
Missense3D af-f1lqh2-f1 modeling (Ittisoponpisan et al., 2019) of the NFKB1 protein resulting in structural damage detection (steric clash), following a substitution of Asp by Glu at residue ID:966.

The substitution (missense) of the nucleotide A by T, for SNP NFKB_85, results in the translation into an aspartic acid instead of a glutamic acid at position 963/970 (or 1,004/1,011), and this in a C-terminal amino acid motif (Figure 7A) which remains identical in NFKB1-201 or NFKB1-202. It would be at the linear end of the protein according to the UniProt (Consortium, 2022) F1LGH2 model of 970 aa. This protein can also be visualized in PeptideAtlas (Desiere et al., 2006) in the D3ZN11 group, with this terminal pattern (958>MPHNYGQDGPIEGKI) previously identified in other observed peptides. After uploading the sequence to the AlphaFold website (Jumper et al., 2021) for 3D structure prediction, the variation was submitted in Missense 3D (Ittisoponpisan et al., 2019) which detected a structural damage (af-f1lqh2-f1-model_v4; residue ID:966; Variant Asp > Glu) (Figure 8). Actually, this substitution triggers a steric clash alert (i.e., number of bad atom–atom overlaps ≥0.4 Å per thousand atoms) and displays a MolProbity score (Davis et al., 2007; Williams et al., 2018) for standard rats of 37.27 (poor structure) and a score for resistant rats of 60.98. Here, a substitution is regarded as damaging if the MolProbity clash score is over 30 and the increase in local clash when compared to the local clash score of the standard structure is over 18. Hence, such a variant, currently assigned as neutral, may subsequently prove to be disease-associated. Although clashes are the single most powerful diagnostic for many kinds of local fitting problems, crystallography studies should be performed in order to assess the impact of such a structure on the function of NFKB1.

A larger biosequence analysis using profile hidden Markov Models (LRDNDSVCDSGVETSFRKLSFSESLTGDGPLLSLNKMPHNYGQDGPIEGKI) was performed with HMMER Web version 2.41.2. It identifies (100%) this sequence as a NFKB p105 subunit fragment in rats and other species. Actually, NFKB1 consists of five subunits: p65 (RelA), RelB, c-Rel, and p50 (specific to NFKB1). Unlike the other subunits, NFKB1 is synthesized as precursors which are proteolytically cleaved into p50. The NFKB subunits, which form homo- and hetero-dimers, are kept inactive in the cytoplasm by the inhibitor of KB proteins; phosphorylation and degradation of IκB through activation of the NF-κB signaling pathway leads to the translocation of NF-κB dimers into the nucleus (Ravi and Bedi, 2004). Therefore, the SNP NFKB_85 is located on the C-terminal side not far (28 aa) from a PEST region of p105, which contains the conserved motif SGVET that is related to the inhibitory KB kinase (IKKB) (Salmerón et al., 2001; Beinke et al., 2002). IKK-mediated phosphorylation predominantly promotes degradation of p105 rather than processing. Degradation of p105 releases p50 and other associated Rel subunits to translocate into the nucleus and modulate target gene transcription. Actually, the PEST sequence acts as a signal peptide for protein degradation (Rogers et al., 1986). For maximum efficiency, it is preferable that the IKK binds to the death domain docking site of p105 which lies upstream of PEST (Beinke et al., 2002). At this point, it, therefore, remains difficult to assess the impact of the substitution on NFKB_85, but it should not be denied for all that, considering its proximity to the PEST domain.

Concerning the SNP NFKB_69, the substitution (missense) of the nucleotide C by T leads to the replacement of an aspartic acid by an asparagine at position 824/970 (or 865/1,011), and this is in the folding between two alpha helices (Figure 7B) according to the model of AlphaFold (Consortium, 2022) F1LGH2 of 970 aa. This substitution is located in the p105 death domain (DD) (Park et al., 2007) according to the protein summary of the Ensembl database. After uploading the sequence to the AlphaFold website (Jumper et al., 2021), the variation was submitted in Missense3D (Ittisoponpisan et al., 2019) which did not detect structural damage on the general model (af-f1lqh2-f1-model_v4; residue ID:827; Variant Asp > Asn) nor on a more specific p105 model focused on the death domain (af-q63369-f1-model_v4; residue ID:376; Variant Asp > Asn).

Some studies link heterozygous mutations in NFKB1 to damages related to immunologic dysfunction (Kaustio et al., 2017). We seem to be in this case, but unfortunately, we do not know if these substitutions linked to this resistant phenotype promote or, on the contrary, alter the specific activity of NFKB1. For example, we do not know if a substitution at SNP NFKB_69 of C instead of T in the death domain enhanced the degradation of p105. This remains to be studied more specifically.

### Multi-level regulation

The multiplicity of substitutions in the SNPs identified in the resistant strain compared to the standard strain involves different structural levels of the cell and its proteins; additionally, it influences different transduction levels of information.

The substitutions identified involve two genes encoding key constituents of the innate immune response, namely, MyD88 and NFKB1. In the information transmission network at the cellular level, the first element MyD88 is attached to the immune receptor, for example, TLR-4, on the cytosolic side of the membrane. It is located upstream of the second key constituent identified in this study: NFKB1. Schematically, NFKB1 has the role of propagating and regulating, integrating *de novo* with the help of other modulators, the immune information a little further toward the cell nucleus in order to ensure appropriate expression of many other genes.

Concretely, these two missenses identified for NFKB1 imply a structural change of the protein complex, which will have consequences on the integration of information. In this study, we demonstrate that integration is doubly modified insofar as this structural modification is also linked to SNPs involving pre- and post-transcriptional regulatory elements, i.e., 5′UTR and 3′UTR, of NFKB1 but also MyD88.

Taken independently, these SNPs have silent consequences as the standard’s Wistar strain is not considered a model of “disease,” but the combination highlighted here (MyD88_50-T, _49-A, _97-C nor NFKB_85-T, _69-T, _45-T) seems to confer particular resistance to DCS to the animals in our study, at least in part. It is, however, interesting to recall that these six identified SNPs are probably not the only ones to confer resistance, insofar as we used the candidate gene approach in this study. Broadening the research seems necessary. Nonetheless and independently from any preconditioning strategy or diving acclimatization, this DCS-resistant animal breeding program resulted in a strain of rats that is three times less likely to develop signs suggestive of DCS (Lautridou et al., 2017; Lautridou et al., 2020).

This model of resistance to DCS makes, therefore, possible the study of its physiological background linked to a fecal metabolome fingerprint (Vallee et al., 2021), which is now associated to a genetic patrimony which differs from that of standard non-selected Wistar rats. The main result of this analysis is a difference between the standard and DCS-resistant rats with respect to this immune-related haplotype. Thanks to this genetic component, this is the first time that an upstream and direct role of the immune response in DCS has been demonstrated. This is of paramount importance because it could change the perception of the evolution of DCS and therefore its therapeutic management. Current therapy is essentially focused on the role of the bubble in the bloodstream, postulating that hyperbaric oxygen therapy can reduce the size of circulating bubbles on one hand and promote oxygenation of ischemic tissues (arguing of a gas embolism) on the other hand. Thus, we hope this study can emphasize the inflammatory effect which is very well-described in the DCS. This also opens the question of the consequences of hyperoxic exposures in diving and also of the necessity and effectiveness of hyperbaric oxygen therapy.

Without being exhaustive, we must also remember that previous studies, one of which involves this same strain of rat, evoke reshuffles at the energy level and therefore mitochondria (Blatteau et al., 2018; de Maistre et al., 2020; Vallee et al., 2021; Boussuges et al., 2022; Desruelle et al., 2022), whereas the mitochondria is itself a major player in the immune response. These studies also propose the idea of a reorganization of the microbial communities (de Maistre et al., 2016a; de Maistre et al., 2016b; de Maistre et al., 2018; de Maistre et al., 2020; Vallee et al., 2021; Desruelle et al., 2022), which have two particularities: interacting with the immune system (Korach-Rechtman et al., 2019) and being sensitive to oxygen (presence of anerobic species). On the other hand, the immune reaction to oxidative stress has been regularly evoked in diving (Thom, 2009; Morabito et al., 2011; Thom et al., 2012; Eftedal et al., 2013; Wang Q. et al., 2015; Thom et al., 2015; Blatteau et al., 2018), and current therapy relies on high-dose oxygen. This opens the question of the consequences of hyperoxic exposures in diving and also of the necessity and effectiveness of hyperbaric oxygen therapy.

Finally, it was shown that the NFKB pathway is implicated in the adaptive response to decompression injuries by modulating the expression of inflammatory factors such as IL-1 beta, IL-6, or TNF-alpha (Wang H.-T. et al., 2015; Wang et al., 2018).

### Moderation, study limitation, and possible future directions

Analyses used in population genetics have been succinctly carried out in order to describe our populations, but it is important to remember that they serve here to essentially confirm the selection process of this population with a low number of individuals rather than to put forward a panximic model which cannot be given by the breeding method of the resistant strain.

It must be remembered that this work has not studied the whole genome and that it is possible that other variants which were not analyzed in this study may play a determinant role. In the same way, silent variants have been discarded from our prior selection, but it is now known that they can influence the overall expression of a gene when another regulatory protein expression uses the area of the SNP, in as a reading window.

Although our results highlight differences between this strain of DCS-resistant rats and standard animals, we have not yet experimentally questioned the relationship between these polymorphisms and direct resistance to DCS: no animal underwent hyperbaric exposure in this study since we used the offspring of DCS-resistant rats, which also serves to confirm the hereditary aspect and therefore the genetic transmission of the characteristics. Although it seems unlikely to us, one could objectify that these differences are not directly related to DCS resistance and that it may represent collateral modifications only. This is the subject of continued experimentation by our research group and others.

Finally, from a clinical point of view, it would be interesting to consider how much the reshuffle of the genotype affects the prognosis of DCS in a short term. As a result, it should be taken into account in DCS therapeutics in order to improve medical care and perhaps start looking at drugs targeting the MYD88/…/NFKB1 signaling pathway (Ramadass et al., 2020), for example, or broaden the questioning of patients to low-noise symptoms that may suggest autoimmune reactions. Ultimately, it also might be worth investigating the whole-genome sequencing analysis of patients presenting with DCS in order to move DCS to more personalized approaches.

## Conclusion

By comparing a standard strain of Wistar rats to a strain of rats resistant to DCS, we identify for the first time a combination of six variants, located on two genes encoding key constituents of the innate immune response, namely, MyD88 and NFKB1.

Four of the six identified variants are located in pre- and post-transcriptional areas, regulating MyD88 or NFKB1 expression. Because of missense mutations, the other two variants induce a structural change in the NFKB1 protein complex. More specifically, the NFKB_85 variant triggers a clash alert for protein structure, according to the modeling by the Missense3D website. Complementary to other studies, this again highlights the importance that the immune response may have in the development of DCS.

## Data Availability

The datasets presented in this study can be found in online repositories. The names of the repository/repositories and accession number(s) can be found in the article/Supplementary Material.
