# Supplementary material for: Highlighting of the interactions of MYD88 and NFKB1 SNPs in rats resistant to decompression sickness: toward an autoimmune response
Source: Front Physiol. 2023 Aug 17;14:1253856. doi: 10.3389/fphys.2023.1253856 (PMC10470123; doi:10.3389/fphys.2023.1253856)

**Supplementary Data**

**Supplementary Data 1: List of primers used in the present study**

Accession number in European Variation Archive

>**TLR-4_rs64613273**

ATGGATATGAGGCCATATCCCAGAAATGCCAGTGCTTGTGTGGTAGAGGCAATTAGATAGGAAGTTCAATGTTATCTTTGACTGTATAGCTAATTTAAAAGAAGACTGGGCTATCTGAAAGCTTGTTTAAAAACAAAATAAATCACGAAGAATGGATGGATAGGTGGATGGTATATTGATTAGCATGTATATGGGAGTTTTACCATCTCATTATTCATCTTTGGAGAGGAGTGGGAACACATACAGTCGAAAAGAATAACATTTTGTGTGATTTTTTACAGGTACTTCCTAATATTACCTACCAATGCATGGATCAGAATCTCAGCAAAATCCCTCATGACATCCCTTATTCAACCAAGAACCTAGATCTGAGCTTCAACCCCCTGAAGATCTTAAGAAGMTATAGCTTCTCCAATTTCTCACAACTTCAGTGGCTGGATTTATCCAGGTAATGAATGAGTTTTATTTGCTGCAGACTGTGAAGTAGTTATTTCTATATCACTGCATTTTTGGCTCAGAAGACCTAGATGTTTCTAAGTAAATTTCCTTTACTCATCTATTCAGTAGACTTAGTCCTTGCTGTAAATCCTTGGGACAGTTACTTTTATTTACCTGGTTTCTRTTTGGTAGGCAGCTAAATAAGAATATCAAAGGGCAGTTCTAACTTCTATATTATTTTAAACTATTTCATTAGGTATTTATGGATCACATATTATATGWTATACATCTAAGCTACAGAACAAAAATTTATAGATACAAAACCCATACTCCTAATTATTAAGCAGGATAAAAATCTTCTTT

>TLR-4_rs64613273-Dir

TCATGACATCCCTTATTCAACCA

>TLR-4_rs64613273-Rev

TACTTCACAGTCTGCAGCAA (ttgctgcagactgtgaagta)

>TLR-4_rs64613273-ProbeC

AAGCTATAGCTTCTCCAATTTCTCACAAC

>TLR-4_rs64613273-ProbeA

AAGATATAGCTTCTCCAATTTCTCACAAC

**>TLR-4_rs107586990**

TGTGTATCAGGTGTATGAATTAAGCAACTCAGGCAAAGAATCATAATCAGCAAAGTTTACTCTTATAAAACCTAAGGAGAGGAGGCTAAGGCCCAGTGAGAACAGAAAGGAACATCATTCTTCTCTGGATCTTTGAATATAAGCACAACATGTAGTGTGCTGCAGTTACCTTAGAAGAGTTTTGATCATTTAAACTGAAGTGAATGTTTCCTTCCTTTCCCTTTTTCTATTGAATATAATTTAAATGGCACTGACTCTTTTTGAGAGACCCTCATTCAAATTTCTTCTTCCATTTTCTGTCAGTTTCTTTTTTTAAATCTAGTTCTACAAGAAATATGACTGATACATGCTCAAAGATATCCTGGTCAATCCTTAGAATGCTATATTTATAAAATAAAAAWTTTTAGTGTACTTTTATTTTTTAAAATCAGTTATTTTTCCTTGCCTTGACTATAAACTCATATCATAAATAAGATTGTCATAGGTATGCTAGGAATGCCCACATTAAGTACAATTTTTCAGAAAGTGTATAAARTATACTGTCATTTTGTCACTGGGTGTCAGTCTTAAGTTATTACCTACTAAGTTATGGATGTCACAGAATCAGTGTTAAAAATAATTTGCTTGATAGAAATATTTTTAATCAGAAAAGAAAAGTGCAGGGGAGTGCAGGAACAGAAATCATAACTTGGTCATTTATTCTTGATTATTCCAGAGCCATAGAAGCTCACACAACTAGATAGTGAGACTAGATAGGCTGACATTGAGGTTCCTTCTCAGCAAGGATACTGTCTGACGGAC

>TLR-4_rs107586990-Dir

AAATCTAGTTCTACAAGAAATATGACTG

>TLR-4_rs107586990-Rev

TTTATGATATGAGTTTATAGTCAAGGCAAG (cttgccttgactataaactcatatcataaa)

>TLR-4_rs107586990-ProbeA

TGCTATATTTATAAAATAAAAAATTTTAGTGTACT

_>_TLR-4_rs107586990-ProbeT

TGCTATATTTATAAAATAAAAATTTTTAGTGTACT

**>MyD88_rs197705412**

CTTACCTGGAGACAGGCTGAGAGCAAACTTGGTCTGGAAGTCACATTCCTTACTTTGCAGGTAATCGTCAGAAACAACCACCACCATGCGACGACACCTGAGGGAGAGTGGCAGAGTTCAGACATTGCAGTGACTTCCTTCAGAGGATGGTGTGGTCAGGAAGGACCACATTACCCAGGCTGGACAGAGCCCAGGTAGCAGGAGAGTCCTAGTATCAGGGCCTCTGGGTTTCATGCACTCAGTCACTCTTAGATGTCTAACCAACCTTTTCTCAATGAGCTCGCTGGCGATGGACCACACGCAGGTGCCAGGCAGGACATCGCGGTCGGACACACACAATTTAAGCCGATAGTCTGTCTGTTCTAGTTGCCGGATCATCTCCTGCACAAACTCAATATCWYTGGGGCAGTAGCAGATGAAGGCGTCGAAAAGCTCCGGCGTTTGCCCTGAGGACGGGGGATGAGGGAAACAATCAGCCTTCTATGGAAGGCTAGAAGGAGCCCAACCTGGAGCCTTCCGTTTAGGGAACCTGGGCCTGCATCCATCCCAAAGCGATGTCATAGCCACGCCCATCCGGCCAGTGAAAAGATAACTCCCTCCCAGATGAAAGCCAGGCTAACAATGGAGTAGACGGGGGCAGTACGGGCTCAGAACCACTCTGAGACAGGACTGCTATTATCCCCATTTCCTAGATGGGGAAACCGAGGTTCAGAAYGAGCGGCTTGCCCAARGTCCCCAGATCCATCCATCCACTTCCAGTATCCATGCTCTCTGCACACCACAAGTCTGGAGCTGAACCC

**>MyD88_rs107423690**

CTTACCTGGAGACAGGCTGAGAGCAAACTTGGTCTGGAAGTCACATTCCTTACTTTGCAGGTAATCGTCAGAAACAACCACCACCATGCGACGACACCTGAGGGAGAGTGGCAGAGTTCAGACATTGCAGTGACTTCCTTCAGAGGATGGTGTGGTCAGGAAGGACCACATTACCCAGGCTGGACAGAGCCCAGGTAGCAGGAGAGTCCTAGTATCAGGGCCTCTGGGTTTCATGCACTCAGTCACTCTTAGATGTCTAACCAACCTTTTCTCAATGAGCTCGCTGGCGATGGACCACACGCAGGTGCCAGGCAGGACATCGCGGTCGGACACACACAATTTAAGCCGATAGTCTGTCTGTTCTAGTTGCCGGATCATCTCCTGCACAAACTCAATATCWYTGGGGCAGTAGCAGATGAAGGCGTCGAAAAGCTCCGGCGTTTGCCCTGAGGACGGGGGATGAGGGAAACAATCAGCCTTCTATGGAAGGCTAGAAGGAGCCCAACCTGGAGCCTTCCGTTTAGGGAACCTGGGCCTGCATCCATCCCAAAGCGATGTCATAGCCACGCCCATCCGGCCAGTGAAAAGATAACTCCCTCCCAGATGAAAGCCAGGCTAACAATGGAGTAGACGGGGGCAGTACGGGCTCAGAACCACTCTGAGACAGGACTGCTATTATCCCCATTTCCTAGATGGGGAAACCGAGGTTCAGAAYGAGCGGCTTGCCCAARGTCCCCAGATCCATCCATCCACTTCCAGTATCCATGCTCTCTGCACACCACAAGTCTGGAGCTGAACCC

>MyD88_rs197705412_rs107423690-Dir

GGTCGGACACACACAATTT

>MyD88_rs197705412_rs107423690-Rev

GCTCCTTCTAGCCTTCCATA (tatggaaggctagaaggagc)

>MyD88_rs197705412_rs107423690-ProbeAT

ATCATTGGGGCAGTAGCAGATG

>MyD88_rs197705412_rs107423690-ProbeAC

ATCACTGGGGCAGTAGCAGATG

>MyD88_rs197705412_rs107423690-ProbeTT

ATCTTTGGGGCAGTAGCAGATG

>MyD88_rs197705412_rs107423690-ProbeTC

ATCTCTGGGGCAGTAGCAGATG

**>MyD88_rs106998802**

ACCAAAAATGTAGCATCAGTATTGTGTGTGTGGCACTTGGCCATCCAACGGCTGGATGTGTGGGACACCATGATCAAGAGGTAGATAGTGGTCTGAGAACACAGCTCAGACGACAGTTTGCCTAACATGGATCCTGAGCTCAATCCCTGACACTGCAYACCAGAGCCAGGACATGGTTTAACCTAAGAAGGACTCAGGCTGCTGCAGGGTGAAAGCTTCTGGAGCTGATGATACCTTCGGCTCCACTTGGTCCCTGCTGAAGCCGAGCCCATGAGACTGAGGCTCAGCCATCGGGGAGCCCTCGAGGACACCACATTATAGAGAAGGTTCTCTTACATGAGAAGTTCCTAACCCAGTTCTCAGCTTCCAAACACCTTAACACACATACAGTCACGAGTATRCTTTCTCAAAATAGAATACTTTTTATTATAAATTTCACATACAGAAGTACAAACCACAAATAGGAGCCTCTCTATTGACATCCTCAGAAAACCTATAAAACAGGTACAGGAGACACTTTCCCAAGGCTGCTTTCAAATGCTCAACATCAAGCATTGAAATGCCCCACGAGCTCGTGCAAAGAGGCCTCCATTCCTCCTCCAGACACTGAGGGGAGACCCATTTTCTTTAGGACTCAGGACCCTGGGGTGTGTGCCCTGAGAGGGACCATGACATTGTCTCTGTGTTAGAGAACTTGAGAGGAATTTGCAAACCGCACTGCTGGGGAGAAAACAGCTGATCCTGCAGCTGGTTTGTGGGGTGAAGCCAAACTGCTTCTCCTTTTTTTTTTAAATCTTCAGT

>MyD88_rs106998802-Dir

CCCAGTTCTCAGCTTCCA

>MyD88_rs106998802-Rev

GCATTTGAAAGCAGCCTTG (caaggctgctttcaaatgc)

>MyD88_rs106998802-ProbeG

GAAAGCATACTCGTGACTGTATGTGTGTTA (taacacacatacagtcacgagtatgctttc)

>MyD88_rs106998802-ProbeA

GAAAGTATACTCGTGACTGTATGTGTGTTA (taacacacatacagtcacgagtatactttc)

**>MyD88_rs107425550**

AGCTATTCTTAGGAGCTGTCCCAAAGGAAACACACATATGCAGATGCCTGTGTGTTCCCGGAAGCACAAACAAGACTATATGGGGCCACATGCCTCCAGCCACTGGGGGCGGAATGTTTTTGTGTGTCTGCTGCTTGTTAACAAAGCAAGTAATCATCGTACAGAAGGTTGGTAGAAAGCCTTCGGGGTTGGCGAGCCAGGGAGGAAGCTTCCCTGATGACATTGTCCTTGTGGTCCTGGTGGCCCAGCTCGTAAGTGATAGGCATGTCAGGGGAGACAGGATGCCACCTCAAGCAAGGCAAAACTGAGATGTGTGCCCAGGCATGGGTGGGTGGGAGTAAAAGGCTGTCCTGGTTGCTCAGGCCAGTCACCACTGAACATGGACTGAGCCTGAGGGGAGYCAAAGATGCAGACAGGACAGCGTTGGAGAATCMGGCTCCAAGTCAGCTCATCTTCCTCTCTGCCCTGTGGGAGGAGGAAAGGGAGTTTAAGGTGGACAGGGGTAAGCAGCTGCCCTGGCAGTCTGTGATCCCTTTAACAGCCAGTGTGGTCCTTCTGCTCCCCTTCAGCACAGACCCCTAGGATAGCGGGAACAGTGCCTCGCCATTTCCCTGCCTGATCTCCATATCCTATTTCTTAGGTGAAGACGCTGAGGCCAAAGATGACTCCTCCACTTAGACTCATTCAGCCCCAATAAACACTGTGCTGCCTGGCAACCCGGCAAGTAACAAGGTCACTTGCAGTCACTGTTCCATAGGACATAAAGATGGTGTCCAGAGGATGTCTGGCTACTCTAGATGA

>MyD88_rs107425550-Dir

GCCCAGCTCGTAAGTGATA

>MyD88_rs107425550-Rev

TTCTCCAACGCTGTCCT (aggacagcgttggagaa)

>MyD88_rs107425550-ProbeC

AGGGGAGCCAAAGATGCAG

>MyD88_rs107425550-ProbeT

AGGGGAGTCAAAGATGCAG

**>MyD88_rs106151549**

ACATATGCAGATGCCTGTGTGTTCCCGGAAGCACAAACAAGACTATATGGGGCCACATGCCTCCAGCCACTGGGGGCGGAATGTTTTTGTGTGTCTGCTGCTTGTTAACAAAGCAAGTAATCATCGTACAGAAGGTTGGTAGAAAGCCTTCGGGGTTGGCGAGCCAGGGAGGAAGCTTCCCTGATGACATTGTCCTTGTGGTCCTGGTGGCCCAGCTCGTAAGTGATAGGCATGTCAGGGGAGACAGGATGCCACCTCAAGCAAGGCAAAACTGAGATGTGTGCCCAGGCATGGGTGGGTGGGAGTAAAAGGCTGTCCTGGTTGCTCAGGCCAGTCACCACTGAACATGGACTGAGCCTGAGGGGAGYCAAAGATGCAGACAGGACAGCGTTGGAGAATCMGGCTCCAAGTCAGCTCATCTTCCTCTCTGCCCTGTGGGAGGAGGAAAGGGAGTTTAAGGTGGACAGGGGTAAGCAGCTGCCCTGGCAGTCTGTGATCCCTTTAACAGCCAGTGTGGTCCTTCTGCTCCCCTTCAGCACAGACCCCTAGGATAGCGGGAACAGTGCCTCGCCATTTCCCTGCCTGATCTCCATATCCTATTTCTTAGGTGAAGACGCTGAGGCCAAAGATGACTCCTCCACTTAGACTCATTCAGCCCCAATAAACACTGTGCTGCCTGGCAACCCGGCAAGTAACAAGGTCACTTGCAGTCACTGTTCCATAGGACATAAAGATGGTGTCCAGAGGATGTCTGGCTACTCTAGATGACTAGGGACAAGCTCCCCCCCTACAACATCAGAG

>MyD88_rs106151549-Dir

CCAGTCACCACTGAACAT

>MyD88_rs106151549-Rev

GGGATCACAGACTGCCA (tggcagtctgtgatccc)

>MyD88_rs106151549-ProbeA

AATCAGGCTCCAAGTCAGC

>MyD88_rs106151549-ProbeC

AATCCGGCTCCAAGTCAGC

**>MyD88_rs198397997**

CGCGACCAACGGTAGAGAGAACAAGTAGGAGTCCACGGACACGGATCCCACGCGGGGGCCTCCCGCAGACATGGCAAGCAACCCTGGGCCCCGGTTCTGTACGGTGCGCTCGCTTGTTGAGCTTCCTACTTCCCTTAAGGCTCCTGCCAACCCCTCCTCCTTTCGCTTTCCGAGAAGCTCCGCCCCTTGCTAGAATCTGGAATCCGGACCCACAACACGCAGCCTGAAATTCAACCCACTTGAGTTCTCACCTCCCCTTGAAGGTGTGAGAGCTTATAGAAACCGCCTCTGACGTCCGCCTTGGCCCACCGACCTGTCCTGGGCGATCTGGTAGTGGTGGCTATGCGTGGTGAAGTGATTCAGCCTTGTTTAGGACTTGACAGGAGTTACAGCTCTTCCGYACAGTAATTTAGATTCTGGTAATAGGGAGGGAAGAGACGGACTGGCTTCGTAAGCAGTAACCTGAAGGCTCWGTCTACCTTAGCCATCTGGGACAAGTGGTGACYGTGCTTTTTAAAGGGAAAGTCCACGACCTTGCCCGAGAATAGACATTTAGGGCATCCTGTCCTTTGTTATCTTAGACAGACAACACTACCTTCCGAGGGTCTGCAAAAAGCACACGGACCTATCACTTTACTCTTTAACGTTGGGCTTAGACCAAGGAAGAGTGTTTTCGTTATTTCGCTCGGTTAATTTACTTAGGTGTCAATCACCAGTACTAGATCTCCCCCTTCTCCTCCCCCGGGCTTAGTAGAATTCCTTGGGGAAAGTGGGCATTAATGGCGTTATCCCAAACGAACG

>MyD88_rs198397997-Dir

GCTTATAGAAACCGCCTCTGA

>MyD88_rs198397997-Rev

TCCCTCCCTATTACCAGAATC (gattctggtaatagggaggga)

>MyD88_rs198397997-ProbeT

AGTTACAGCTCTTCCGTACAGTA

>MyD88_rs198397997-ProbeC

AGTTACAGCTCTTCCGCACAGTA

**>NFKB1_rs198613385**

ACTGTACTGTAGATGGCTAGAAAGAACACCAGGTGGGGACTGCGATACCTTAATGACAGCGGGGACCCAGCAGAGGGACAGCAGTGACAACACAAAGCAAGTGTAATCCAATAGCAGCTGGAAAAGCTCAAGCCACCATACCCCAAGCCACACCGAGTGCAGCCGTGATTGCTAGACACCATCTGTATATGACAACTAAATTTCATTTCAGAGCCAAGAAAGGAAGCCAAATTCGGAAGGCCTCGAATGACATCAAGATTGACCCTGCAGGCCGGTGACCTTCCAGCACCTTGGGATGCGTTTTTTGCCTTCTCCTTCGGAACGATATGATGGCCTTTCAGGGCTTTGGTTTACAGTGTGGGGAACCGCCAGCAGGCTAAATTTTGCCTTCTATAGGTCCWTCCTGCCCATAATTGTGAGGCATTTTGTTCAGAGATAGCAGTGGGCCATCTCCAGTGAGGGACTCCGAGAAGCTGAGTTTGCGGAAGGATGTCTCCACACCACTGTCACACACGCTGTCATTATCTCGGAGCTCATCTGAGGAGAAAGCACAGATTCATCAGGGGCTGAGCTAGGCCATGGCGAGGCTTTCGTTCTGCCTGCTGTCCTCGTGCCCTTCAACTGTCAACCTCACACTGTCTAGAATCACCTGCAGAAGGAGGTGCAAATGAGGGACCAGCCAGATGAGACTGGTGTGAGGGCATGTCTGAGGGGACGGTCCTGACAGTTAACCGATGTGGGCCACTGTAGACAGCACAGTCCCGAGCTGTGTAAGAAAGCGAGCTCAGCGTGAGCCKCGAG

>NFKB1_rs198613385-Dir

CCTTTCAGGGCTTTGGT

>NFKB1_rs198613385-Rev

GCAAACTCAGCTTCTCGG (ccgagaagctgagtttgc)

>NFKB1_rs198613385-ProbeA

TAGGTCCATCCTGCCCATAATTGTGAG

>NFKB1_rs198613385-ProbeT

TAGGTCCTTCCTGCCCATAATTGTGAG

**>NFKB1_rs197284969**

GAGCACACCGATCACAGCTTGGCGGGATCCCCATCWTGTGGGCAGTAGTCGTTTTTACAAGAAGGMTCTGAGAATTTGAAAACGGGGAATGCCTAGAGACAGGTAATCAGTCTTCTCAGGAAAACTGGCGTCATCCTGTCCTGGCCTAGGTCCCTTTGCCTCCCATGCCCCAGGCAACGACTGGTTTCCTTCCACAAAGTTACAACGCAGGGGAGAGGGCTGTGCCGCTCAGTGGTGTTGGCACAGTCAACTACCAATGGTTGCRCACAACTCTAAATGGTACGGCGGTGCCTTACCTCATAGTTGTCCATAAGAGTTTTAGAAGGGGCGGGACTCAGCCGGAAGGCATTGTTCAATATCCCCAGACCTAACTTCTGCGCCAGAGTGGCCCAGTTCTTGTYGGGATCAGGAATTTCCAAGAGCTTGCAGAGTTGCAGCCTCGTGTCTTCTGTCAGCTGCTTAATGTCCCCTAGGGAGGAAACGTGACTGCTTTAGCCTTCCYGGGACAATCAGTCTGAGGGGACATGCCATCATCCAACTTCTGATGGAGGCAATCAAGCTTCTACAAAGTCCAGAGGCCCTGCCCAGCAGCAGCAGCTCAGAGCCACKCCTCCAGGGACCTCAGATTCCAAAATAAAAGYGAGAGTGTTCGCAGCACACCCCGGCCCRGGCTGGCTTCTCCTCTCCTCCTGTAAACCACAGCACCGGGCAACAGTGGCTGAGAGAATTTGTTCTGCCACAGCTCACCTTGTGGGAGTATGTCATCAGATGTGAACACTGGCTCATATGGTTTCCCATTTA

>NFKB1_rs197284969-Dir

CCTCATAGTTGTCCATAAGAGTTT

>NFKB1_rs197284969-Rev

AGCTGACAGAAGACACG (cgtgtcttctgtcagct)

>NFKB1_rs197284969-ProbeT

TGGCCCAGTTCTTGTTGGG

>NFKB1_rs197284969-ProbeC

TGGCCCAGTTCTTGTCGGG

**>NFKB1_rs106154760**

GGAATGGTGTCTGGAATGGAATGCTGAYGCCAGGTGTCCAGATGAAAGTGCCTGGGCCAGAATGTGTCAGGTTTGCCCTCAGTCTTGGGGTAGAGCACTTTTGTTTCATAGCTGTCTGTTTGAACATTTCTAGCAGCAAAGGAATGTGTCTAAAGGAGTATGTGGATGTGACAAGCAGGCTGTGGGGTGTTCCCACTGGGACAGGGACTCCTCTGAGAAAGTAGCTGCAGAGACCAGACAAGATGAGGTCAACAGTGTGCTTGTACTTTTATTATAAAAGTAAAATAAAAAATAGCATTTATAAATCCAACATTTCTTCTTTTAAAGTATTTGATTTAGAAAACATATTTACAATAGTAAAATGCAGAAAAGGGGGAAAATACTATTTTCAGCACTGATTRTAGCAGGTTTTAAATATTGTTAAGTTCATGTCTTTTTTTTTTTTAAATCACAGATAAACCACATTAGAAAAAGCCATGTCTTTTTTTTATTGYGCATGAAGTTACTCACTTAAAAATATCTTTTTCCTTAATCTTAATTTGAGTGCATGACTGTACTGTAGATGGCTAGAAAGAACACCAGGTGGGGACTGCGATACCTTAATGACAGCGGGGACCCAGCAGAGGGACAGCAGTGACAACACAAAGCAAGTGTAATCCAATAGCAGCTGGAAAAGCTCAAGCCACCATACCCCAAGCCACACCGAGTGCAGCCGTGATTGCTAGACACCATCTGTATATGACAACTAAATTTCATTTCAGAGCCAAGAAAGGAAGCCAAATTCGGAAGGCCTCGAATGAC

>NFKB1_rs106154760-Dir

ACAATAGTAAAATGCAGAAAAGGG

>NFKB1_rs106154760-Rev

CAGTACAGTCATGCACTCAAATTA (taatttgagtgcatgactgtactg)

>NFKB1_rs106154760-ProbeG

AAACCTGCTACAATCAGTGCTGAAAAT (attttcagcactgattgtagcaggttt)

>NFKB1_rs106154760-ProbeA

AAACCTGCTATAATCAGTGCTGAAAAT (attttcagcactgattatagcaggttt)

**>NFKB1_rs197247545**

AGCACTTTTGTTTCATAGCTGTCTGTTTGAACATTTCTAGCAGCAAAGGAATGTGTCTAAAGGAGTATGTGGATGTGACAAGCAGGCTGTGGGGTGTTCCCACTGGGACAGGGACTCCTCTGAGAAAGTAGCTGCAGAGACCAGACAAGATGAGGTCAACAGTGTGCTTGTACTTTTATTATAAAAGTAAAATAAAAAATAGCATTTATAAATCCAACATTTCTTCTTTTAAAGTATTTGATTTAGAAAACATATTTACAATAGTAAAATGCAGAAAAGGGGGAAAATACTATTTTCAGCACTGATTRTAGCAGGTTTTAAATATTGTTAAGTTCATGTCTTTTTTTTTTTTAAATCACAGATAAACCACATTAGAAAAAGCCATGTCTTTTTTTTATTGYGCATGAAGTTACTCACTTAAAAATATCTTTTTCCTTAATCTTAATTTGAGTGCATGACTGTACTGTAGATGGCTAGAAAGAACACCAGGTGGGGACTGCGATACCTTAATGACAGCGGGGACCCAGCAGAGGGACAGCAGTGACAACACAAAGCAAGTGTAATCCAATAGCAGCTGGAAAAGCTCAAGCCACCATACCCCAAGCCACACCGAGTGCAGCCGTGATTGCTAGACACCATCTGTATATGACAACTAAATTTCATTTCAGAGCCAAGAAAGGAAGCCAAATTCGGAAGGCCTCGAATGACATCAAGATTGACCCTGCAGGCCGGTGACCTTCCAGCACCTTGGGATGCGTTTTTTGCCTTCTCCTTCGGAACGATATGATGGCCTTTCAGGGC

>NFKB1_rs197247545-Dir

AGCAGGTTTTAAATATTGTTAAGTTCA

>NFKB1_rs197247545-Rev

ATCTACAGTACAGTCATGCAC (gtgcatgactgtactgtagat)

>NFKB1_rs197247545-ProbeT

AAGCCATGTCTTTTTTTTATTGTGCA

>NFKB1_rs197247545-ProbeC

AAGCCATGTCTTTTTTTTATTGCGCA

**>NFKBia_rs8150309**

GAGCTGCTTCTACAAATAACTCCCATTCCTGGACTCTCTCCTCCTCAGCCTAAACAGAACGCAGGCGCCGGGCTGCAGGAGTTCCCTTCACCAATTCTCTCCTTTCAAAAGTCTAAGCTATTGCAGGGGACATAGCTCAATGGTAGAACATGTGCCTATTACCACGTGTTAAAGCCTGGGTTCAATTCCCACAACTCCCTCTTACTGCCCCATTYCGAAACCCACAAGGRTAAGCTATTAAAGCAAACCAGAGACACGTTCTTGGGATCCTGGCTTTGCAAGCTGAGACTGAGAAGTTGAATAACTCAGGTCCCTATGATACAGAACAGCTCTAGAACAGACCTAGGTGTGGTGAGGGAGAATGGACCACTCTGGCAGTAATGAACACGCACCTCATCTTYCGTGAATTCTGACTCCGTGTCATAGCTCTCCTCATCCTCACTCTCGGGCAGCGTCTGAAGATTTTCCAGGGTCAGCTGGCCCAGCKGCTGCTGTATCCGGGTACTTGGGCGGCCCCACGTAAGCTGGTAGGGGGAGTAGCCCTGGTAGGTTACTCTGTTGACATCAGCCCCACACTTCAACAGGAGCGAGACCAGGTCAGGATTCTGCAGGTCCACTGCAAGGTGGAGGGCTGTCCGGCCATTACAGGGCTCCTGAAACCAAAGGCAATCAGAAGTGTTTATCTGGAAATCTGCTCACAGTAAGAGCCAGCCCAGCAGAGTTTAGGAGGAAGGGGAAGTACTCACCTGAGCGTTGACATCAGCACCCAAAGTCACCAAGTGCTCCACGATGCCCAGGTAG

>NFKBia_rs8150309-Dir

AGCTGAGACTGAGAAGTTGAATAA

>NFKBia_rs8150309-Rev

AGGATGAGGAGAGCTATGACA (tgtcatagctctcctcatcct)

>NFKBia_rs8150309-ProbeT

TGAACACGCACCTCATCTTTCGT

>NFKBia_rs8150309-ProbeC

TGAACACGCACCTCATCTTCCGT

**>NFKBia_rs8168900**

TTCACCAATTCTCTCCTTTCAAAAGTCTAAGCTATTGCAGGGGACATAGCTCAATGGTAGAACATGTGCCTATTACCACGTGTTAAAGCCTGGGTTCAATTCCCACAACTCCCTCTTACTGCCCCATTYCGAAACCCACAAGGRTAAGCTATTAAAGCAAACCAGAGACACGTTCTTGGGATCCTGGCTTTGCAAGCTGAGACTGAGAAGTTGAATAACTCAGGTCCCTATGATACAGAACAGCTCTAGAACAGACCTAGGTGTGGTGAGGGAGAATGGACCACTCTGGCAGTAATGAACACGCACCTCATCTTYCGTGAATTCTGACTCCGTGTCATAGCTCTCCTCATCCTCACTCTCGGGCAGCGTCTGAAGATTTTCCAGGGTCAGCTGGCCCAGCKGCTGCTGTATCCGGGTACTTGGGCGGCCCCACGTAAGCTGGTAGGGGGAGTAGCCCTGGTAGGTTACTCTGTTGACATCAGCCCCACACTTCAACAGGAGCGAGACCAGGTCAGGATTCTGCAGGTCCACTGCAAGGTGGAGGGCTGTCCGGCCATTACAGGGCTCCTGAAACCAAAGGCAATCAGAAGTGTTTATCTGGAAATCTGCTCACAGTAAGAGCCAGCCCAGCAGAGTTTAGGAGGAAGGGGAAGTACTCACCTGAGCGTTGACATCAGCACCCAAAGTCACCAAGTGCTCCACGATGCCCAGGTAGCCATGGATAGAGGCTAAGTGTAGACACGTGTGGCCTGCAGGATATAAGACGGAACTGTAACCACTTGTTTCAATCCTCACACAAGT

>NFKBia_rs8168900-Dir

GTGAATTCTGACTCCGTGTCATA

>NFKBia_rs8168900-Rev

GGCTGATGTCAACAGAGTAAC (gttactctgttgacatcagcc)

>NFKBia_rs8168900-ProbeG

CAGCAGCCGCTGGGC (gcccagcggctgctg)

>NFKBia_rs8168900-ProbeT

CAGCAGCAGCTGGGC (gcccagctgctgctg)

**>NFKBia_rs106621084**

TAAGAGCCAGCCCAGCAGAGTTTAGGAGGAAGGGGAAGTACTCACCTGAGCGTTGACATCAGCACCCAAAGTCACCAAGTGCTCCACGATGCCCAGGTAGCCATGGATAGAGGCTAAGTGTAGACACGTGTGGCCTGCAGGATATAAGACGGAACTGTAACCACTTGTTTCAATCCTCACACAAGTCACGTCTCCTTTCTTGGGGACAACCACAAACAACCTCAATTTTGAAGAACTCAAAAGTCTGGGTTCTCAGTGAACGTCATGTAGGAATCATGGAGAAGACTTTTTAAAAGTCCACACTGCCTGAACTTCTTATACGTTGAGGCGTTTTAGCTCCAGGCTAGACTCAAGGGGACCCGTCTCTCCCTTCTCTGCGTCCCTGGATGGACTGGCAGACYTACCGTTGTAGTTGGTGGCCTGCAGGACGGAGTGGAGATGCTGGGGCGTACAGGTCTGAGTCAAGACTGCTACACTGGCCAGGCAGCCCTGCTCACAGGCAAGATGGAGAGGGGTATTTCCTCGAAAGTCTCGGAGCTCAGGATCACAGCCAGCTTTCAGAAGTGCCTCAGCAATTCCTGGCTGGTTGGTGATCACAGCCAAGTGGAGTGGAGTCTACGAATGGAAGAGGAAACAGAAAGAGCCTAAGCCATGGAGCAAACCCATAGTCTTGAGTTTCCTGAACCCCAGACACCTCCTATTACAGAATCCCCTCCTCCCATTTGTCTACAGGAGATCAGGCAAACAGGAGGCCCTACGTGGGTCATAAAGACCTCAACCAACCCTTGGGATTTTCCTTCT

>NFKBia_rs106621084-Dir

GTCCACACTGCCTGAAC

>NFKBia_rs106621084-Rev

CCCAGCATCTCCACTCC (ggagtggagatgctggg)

>NFKBia_rs106621084-ProbeT

GGACTGGCAGACTTACCG

>NFKBia_rs106621084-ProbeC

GGACTGGCAGACCTACCG

**>NLRP-3_rs106539953**

AGCGCTTCCTAGGTAAGCGCTCTACCACTGAGCTAAATCCCCAGCCCCAATCATAATTCTTAATGTTCCTCCTGTAACAGCACAGKCCTGCTGACCGCTTATTGTTGGGGAATAGATGTGTGGGCTCTTCCTGGATTCTGCCTTTCTGTCTCTGCTGCAGATTACTGTAAGATATACAGAAGGCATGTGAGAAGCAGGTTCTACTCCATCAAAGACAGGAATGCACGTCTAGGTGAGAGCGTGGACCTCAACAGACGCTACACCCAGCTCCAACTGGTCAAGGARCATCCAAGCAAGCAGGAAAGGGAGCATGAACTCTTGACCATTGGCAGGACTAAGATGTGGGACAGRCCCATGAGCTCCCTTAAGCTGGAGCTGCTCTTTGAGCCTGAGGATGAACRCTTGGAGCCCGTGCACACAGTGGTGTTCCAGGGAGCAGCAGGCATCGGAAAAACAATCCTAGCCAGGAAGATTATGTTGGACTGGGCCTTGGGGAAGCTCTTCAAAGACAAATTTGATTATTTGTTCTTTATCCACTGTCGAGAGGTGAGCCTCAGGGCACCAAAGAGCCTAGCAGATCTCATTATCAGCTGCTGGCCTGACCCAAACCCACCAGTCTGCAAGATCCTGTGCAAGCCTTCCAGGATCCTCTTCCTCATGGACGGCTTTGATGAGCTGCAGGGGGCCTTTGATGAGCACATCGAGGAGGTCTGCACAGACTGGCAGAAGGCTGTGAGGGGAGACATTCTGCTAAGCAGCCTCATCCGAAAGAAGTTGCTGCCTAAGGCCTCTCTGCTTATT

>NLRP-3_rs106539953-Dir

TTGACCATTGGCAGGACT

>NLRP-3_rs106539953-Rev

TAATCTTCCTGGCTAGGATTGT (acaatcctagccaggaagatta)

>NLRP-3_rs106539953-ProbeG

CGGGCTCCAAGCGTTCAT (atgaacgcttggagcccg)

>NLRP-3_rs106539953-ProbeA

CGGGCTCCAAGTGTTCAT (atgaacacttggagcccg)

**>NLRP-3_rs197296817**

TCTGTCTGGGCTGAGCATTGAAAGACTGTGAGTGTTTGTTGCTCTGCCATTACTATAGCTGATTCTGCTGGCTCCCTCCTTCCTTTGTTCCTTCCCGTCAGATGAAAATAGAAGCAAGAAACCAGAGCCTTCCATTCAAAGAAATCGGGAAGCAATCTCTTCTGCTTTCTCAGTGACTGAAGGAGGAAGCTGAGGTTGAGAAACTGTGAAGGCCATGTCCAGGTCCCAGACTCATCACCATGGGTTCTGGTCGTACACGGGTCCCGGTGACCTTGTGTGTGCTTGTTCTCTGCATGCCGTATCTGGTTGTGTTAATGCCTTTCTTCTTTTTAATTTCTACAGAGGAACTTTGCTTCCATGGCTCAGGACATGATCCTGGATCAAGCTAAGAGAACTTTCTRGGAAGCCTTTAGCTTGCTCAGCTCTTCCTAAGTCAGAGATGACGATTCACAGAATCAAGTCGTAGTTTTTTTCCTTTGAGTTTTTCGAGTTAAGAAACTTTAAAAACTGTGCCAGTCTATGGGGTACCCTGAGATGTTTCAATATTAGCATCTAGTTTCCTGGATTTCTGCTAACGCCGTTTTCCGGAAGTGGAGAYTTTTGACTCTTTTTTTACTCACACGGAGTTTTCATTCCTGCTCTGCCAGCGTGGATCTAAGCCCAAAGACCCTCGCAAGAGGTGCTGATGAAGATGATGAGTGTTCGCTGCAAGCTGGCCCAGTATCTAGAGGACCTGGAAGATGTGGACCTCAAGAAATTTAAAATGCATTTGGAAGATTACCCACCCGAGAAAGGCTGTGT

>NLRP-3_rs197296817-Dir

CTTCCATGGCTCAGGAC

>NLRP-3_rs197296817-Rev

CCAGGAAACTAGATGCTAATATTG (caatattagcatctagtttcctgg)

>NLRP-3_rs197296817-ProbeG

AAGGCTTCCCAGAAAGTTCTCT (agagaactttctgggaagcctt)

>NLRP-3_rs197296817-ProbeA

AAGGCTTCCTAGAAAGTTCTCT (agagaactttctaggaagcctt)

**>NLRP-3_rs198158662**

GAGAAACTGTGAAGGCCATGTCCAGGTCCCAGACTCATCACCATGGGTTCTGGTCGTACACGGGTCCCGGTGACCTTGTGTGTGCTTGTTCTCTGCATGCCGTATCTGGTTGTGTTAATGCCTTTCTTCTTTTTAATTTCTACAGAGGAACTTTGCTTCCATGGCTCAGGACATGATCCTGGATCAAGCTAAGAGAACTTTCTRGGAAGCCTTTAGCTTGCTCAGCTCTTCCTAAGTCAGAGATGACGATTCACAGAATCAAGTCGTAGTTTTTTTCCTTTGAGTTTTTCGAGTTAAGAAACTTTAAAAACTGTGCCAGTCTATGGGGTACCCTGAGATGTTTCAATATTAGCATCTAGTTTCCTGGATTTCTGCTAACGCCGTTTTCCGGAAGTGGAGAYTTTTGACTCTTTTTTTACTCACACGGAGTTTTCATTCCTGCTCTGCCAGCGTGGATCTAAGCCCAAAGACCCTCGCAAGAGGTGCTGATGAAGATGATGAGTGTTCGCTGCAAGCTGGCCCAGTATCTAGAGGACCTGGAAGATGTGGACCTCAAGAAATTTAAAATGCATTTGGAAGATTACCCACCCGAGAAAGGCTGTGTTCCGATCCCCAGGGGCCAGATGGAGAAGGCAGATCACTTGGATCTAGCCACACTCATGATTGACTTCAATGGGGAGGAGAAGGCGTGGGGCATGGCTGTGTGGATCTTTGCAGCGATCAACAGGCGAGACCTCTGGGAAAAAGCTAAGAAGGACCAGCCAGAGTGGAGTGAGTGAGAAGGTTTTTTTTTTTTTTCCT

>NLRP-3_rs198158662-Dir

GGTACCCTGAGATGTTTCAAT

>NLRP-3_rs198158662-Rev

CATCATCTTCATCAGCACC (ggtgctgatgaagatgatg)

>NLRP-3_rs198158662-ProbeT

GAGATTTTTGACTCTTTTTTTACTCACACGG

>NLRP-3_rs198158662-ProbeC

GAGACTTTTGACTCTTTTTTTACTCACACGG

**>NLRP-3_rs106860510**

AAATTGTGAAACAAAACGTACCTTAGAAGCTCTCCAGGAGGAAAAGCCTGAGCTGACTGTTGTTTTTGAGATTTCTTGGTAGGTGTGGAAGCAGGACTGCCAGGTGCCCCGGTCCCACCCCAAGCCCTACTGTTCTTCAGTGCTGCAAGCCCTCCTTCACCAGCAGGACCAAGCCAGGGCGCTTTTAGTGTAGGGTCTGGAGCAAAGGCCTGTGTGGGAACAAGTATGCTTTCTCCAGGGAAGACACTGATAATACGAAACTACTAGAGGACGCCCTTCCTGTCAGGTCCTCAGTCAGCAAGTTGTTCCCTCTTGGTGACCTGATGGGACCATTTTCTAGAAAATGCATTTTCTGTCCTTTTCCTCCTCTTCCTCCTCCTCTTCTCCCCCACTTCTCTCTYGTTTTCTTGATATGTGTTCAGAGCATTGCTCYTGTCTTCTTGTTAACTGACCATACCATATAAGTAGCTGGCAAGTTTGTGGCAGCCTCTCAGCTACGTCTTTGCTTTTATTTTTATTTTTTTATTTATTTATTTTTTTTTGATTCTTTTTTTCGGAGCTGGGGACCRAACCCAGGGCCTTGCGCTTCCTAGGTAAGCGCTCTACCACTGAGCTAAATCCCCAGCCCCTGTTTTTATTTTTTAATAGTTATGAAAACTCAGAGATTTATAAGATGCATTTTTGTGTTTATTGTATGTTTGTACTGCTTTCTTAATTTAAAAATGTATCTAGAATTCTTTTAAGTTATTTATCCAAACTTCTAAAAATAAATCACTTTAAAATATCTCTTTGCAAAATGAT

>NLRP-3_rs106860510-Dir

TGTCCTTTTCCTCCTCTTCC

>NLRP-3_rs106860510-Rev

GTGGTAGAGCGCTTACCT (aggtaagcgctctaccac)

>NLRP-3_rs106860510-ProbeT

TCTTGTTTTCTTGATATGTGTTCAGAGCAT

>NLRP-3_rs106860510-ProbeC

TCTCGTTTTCTTGATATGTGTTCAGAGCAT

**>UCP-1_rs8146186**

TTTTTTTCAGAGCTGGGGACCGAACCCAGGGCCTTGCGCTTGCTAGGCAAGTGCTCTACCACTGAGCTAAATCCCCAACYCCCCTCCGTTACTTTTATCTTGCTTCGTGTAGTTTATGATTCACGTAAGCTGCCACCGCCACGGCTTTATGACTTTCAAAGAGCTAACGGGATGGTGCCGTGTACTTCCCTAGGTTTGCGCCTTCTTTTCTGCGACTCGGATCCTGGAACGTCATCATGTTTGTGTGCTTTGAACAGCTGAAGAAAGAGCTGATGAAGTCCCGGCAGACAGTGGACTGCACCACATAGGCGACTTGGAGAAAGGGATGCTAAACACCATTGGGCTCCTATGCTGGGCTCCTATGCTGGGAGACCACGAATAAAACCAACCAAAGAAATCARACGAACAGCTCTGCTCACTTTATTTACATTAAAAGATAACTTCTTTAAAGGGAAAACTAATACATACACATAATTTTTATTCTCACTGTCTTAAGGACGTTAGAGCATAGCATCCAGTAATATCCTGTCAAAATAATACCGATATAAGATACTGTATTTATCTGGTCTTTGGGTGGGAGTGTCTGATGGGGTATAAATAATTTTAATTACAGCTCAAACTAGTGGAAAGGAAAAACTAAACCTAAAACACTTTACATCAATCAACACTTAAAAAAGAAGCTATCAAAGAAAATATTGCTAATTCATTTTATTATTATTATTTATTCACAGCTAATATACCCAATAAAGTACTGTTAATTCCATCTACGATTTGTCTTTTTGAGGAGCCTCAATTTAAGAG

>UCP-1_rs8146186-Dir

CGACTTGGAGAAAGGGATG

>UCP-1_rs8146186-Rev

ATGCTCTAACGTCCTTAAGACA (tgtcttaaggacgttagagcat)

>UCP-1_rs8146186-ProbeG

AGCAGAGCTGTTCGTCTGATTT (aaatcagacgaacagctctgct)

>UCP-1_rs8146186-ProbeA

AGCAGAGCTGTTCGTTTGATTT (aaatcaaacgaacagctctgct)

**Supplementary Data 2:** viability of primer extracted from the literature and presence of genetic diversity between the 2 populations studied

**Supplementary Data 3:** *Multiple Correspondence Analysis (MCA) plot conducted on SNPs of MyD88 and NFKB1, strain (Standard or Res), sex (Male or Female) and chromosome (as supplementary category, A or B). Main categories are displayed in black. Observations and their identity are in blue color. Supplementary variables are in grey color. The results of the* *Agglomerative Hierarchical Clustering (ACH), showing the different classes, are reported on the sidelines of the MCA.*


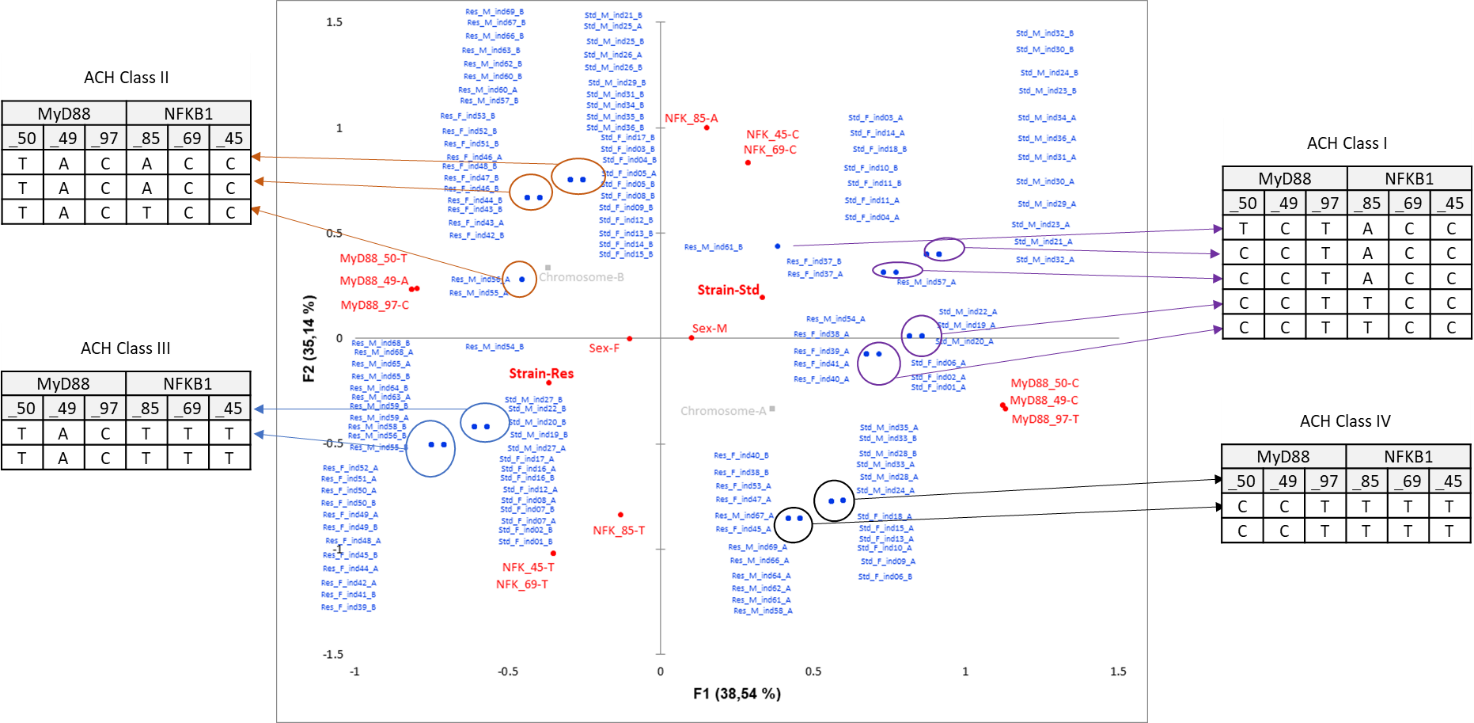


Alleles frequencies according to the ACH


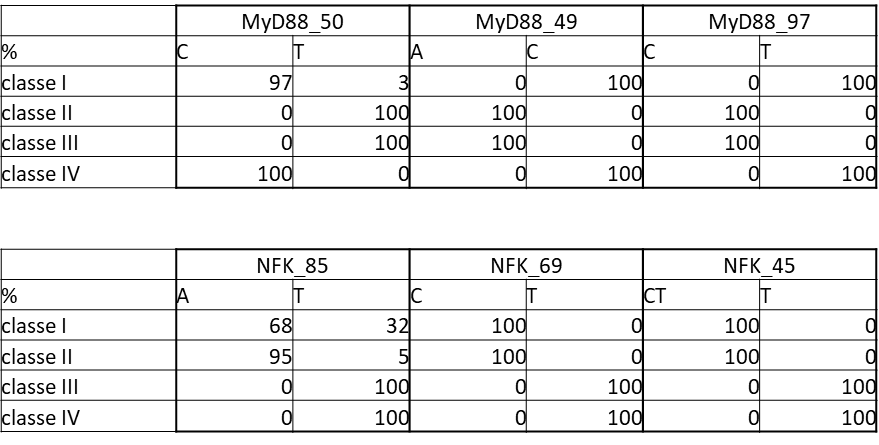

Supplement: Supplementary file 1 [file DataSheet1.docx]
